# Long-chain polyunsaturated fatty acid-containing phosphatidylcholines predict survival rate in patients after heart failure

**DOI:** 10.1016/j.heliyon.2024.e39979

**Published:** 2024-10-30

**Authors:** Aleš Kvasnička, Karel Kotaška, David Friedecký, Karolína Ježdíková, Radana Brumarová, Tomáš Hnát, Petr Kala

**Affiliations:** a Laboratory for Inherited Metabolic Disorders, Department of Clinical Biochemistry, University Hospital, Olomouc, Czech Republic; b Faculty of Medicine and Dentistry, Palacký University in Olomouc, Czech Republic; c Department of Medical Chemistry and Clinical Biochemistry, 2nd Medical Faculty, University Hospital Motol, Prague, Czech Republic; d Department of Cardiology, University Hospital Motol and 2nd Faculty of Medicine, Charles University, Prague, Czech Republic; e Center of Experimental Medicine, Institute of Clinical and Experimental Medicine, Prague, Czech Republic

**Keywords:** Lipidomics, Eicosanoids, Survival rate, Heart failure, PUFA, PC, HF survival, Atherosclerosis, Phosphatidylcholine

## Abstract

**Background:**

Heart failure (HF) is becoming an increasingly prevalent issue, particularly among the elderly population. Lipids are closely associated with cardiovascular disease (CVD) pathology. Lipidomics as a comprehensive profiling tool is showing to be promising in the prediction of events and mortality due to CVD as well as identifying novel biomarkers.

**Materials and methods:**

In this study, eicosanoids and lipid profiles were measured in order to predict survival in patients with de novo or acute decompensated HF. Our study included 50 patients (16 females, mean age 73 years and 34 males, mean age 71 years) with de novo or acute decompensated chronic HF with a median follow-up of 7 months. Lipids were semiquantified using targeted lipidomic liquid chromatography-mass spectrometry (LC-MS/MS) analysis. Eicosanoid concentrations were determined using a commercially available sandwich ELISA assay.

**Results:**

From 736 lipids and 3 eicosanoids, 39 significant lipids were selected (by using the Mann-Whitney *U* test after Benjamini-Hochberg correction) with the highest number of representatives belonging to the polyunsaturated (PUFA) phosphatidylcholines (PC). PC 42:10 (p = 1.44 × 10^−4^) was found to be the most statistically significantly elevated in the surviving group with receiver operating characteristics of AUC = 0.84 (p = 3.24 × 10^−7^). A multivariate supervised discriminant analysis based on the aforementioned lipid panel enabled the classification of the groups of surviving and non-surviving patients with 90 % accuracy.

**Conclusions:**

In the present study we describe a trend in PUFA esterified in PC that were systematically increased in surviving patients with HF. This trend in low-abundant and rarely identified PUFA PC (mainly very long chain PUFA containing PC such as PC 42:10 or PC 40:9 containing FA 22:6, FA 20:5 and FA 20:4) suggests candidate biomarkers.

## Highlights

• Discovery of systematic alterations in the length and saturation of PC acyl chains.
• Low abundant PUFA PC (PC 42:10, PC 36:6, PC 40:9) showed the best clinical performance.
• Multivariate discriminant analysis classified HF patients with 90 % accuracy.

## 1 Introduction

Heart failure (HF) is becoming a significant public health concern, largely due to the ageing population and the associated mortality from de novo or acute decompensated HF. Although the age-adjusted incidence of HF is decreasing in developed countries, probably due to improved management of cardiovascular disease (CVD), the overall incidence is increasing as the population ages [1]. The current prevalence of heart failure is estimated to be 1–2% in all adults and up to 10 % in those over the age of 70 [2,3]. It is likely that the true prevalence of heart failure is higher than that indicated by studies, which usually include only cases that have been recognised and diagnosed. It is therefore beneficial to investigate the diagnosis and progression monitoring of CVD and de novo or acute decompensated HF using novel approaches such as lipidomics, with the objective of gaining insight into the underlying biochemical processes behind and stratifying risk groups.

Lipidomics is a field of study that focuses on the analysis of lipids, including their quantification, as well as the study of their catabolic or anabolic biochemical pathways. The association of changes in the plasma lipidome with the progression of CVD in patients or the prediction of its acute complications has already been well described in the literature. Promising results from recent lipidomics studies have defined several CVD-related lipid classes (mainly ceramides) and individual plasma lipids that are attracting the attention of clinicians, given that they are already part of the Mayo Clinic test catalogue (CERAM test) [[4], [5], [6]]. Ceramides are bioactive sphingolipids with a pivotal role in cell signalling, proliferation, senescence, adhesion, migration, and angiogenesis. They have recently been shown also to influence CVD-related processes, including LDL aggregation and uptake, endothelial dysfunction, and inflammation [7,8]. A ceramide- and phospholipid-based risk score, CERT2, has been developed and validated on multi-cohort samples to effectively predict the risk of cardiovascular events and death in patients with coronary artery disease [6]. Similarly, the CERAM test, which is based on the analysis of plasma ceramides (16:0; 18:0; 24:1 and their ratios to 24:0), can predict the risk of major adverse cardiovascular events (such as myocardial infarction, coronary revascularisation, acute coronary syndrome hospitalization and mortality) within the next 1–5 years [9]. Nevertheless, it would be advantageous to test these findings in a clinical setting with the potential to identify new lipids that could be used to predict the survival of patients with de novo or acute decompensated HF.

Eicosanoids are bioactive lipid mediators which are derived from the catalysis of n-3 and n-6 polyunsaturated fatty acid (PUFA) substrates by lipoxygenases (LOXs), cyclooxygenases (COXs), or cytochrome P450s (CYPs). Epoxyeicosatrienoic acids (EETs) and 20-hydroxyeicosatetraenoic acid (20-HETE) are the products of CYPs epoxygenases (EETs) and ω-hydroxylases (20-HETE), respectively. The subsequent rapid conversion of EETs to the less active dihydroxyeicosatrienoic acids (DHETs) is catalysed by soluble epoxide hydrolases (sEH). EETs exert a variety of physiological functions, including cell proliferation, inflammation modulation, vascular function (vasodilation) and natriuresis. Chronic vascular inflammation may contribute to the development and progression of atherosclerotic cardiovascular disease, including coronary artery disease (CAD) and acute myocardial infarction (AMI). Anti-inflammatory drugs (such as interleukin-6 receptor antagonists) have demonstrated their efficacy as a novel therapeutic approach in CAD [10]. The vasoactive and natriuretic effects of eicosanoids suggesting their potential beneficial role in heart failure have been tested in preclinical studies in various animal models, leading to the development of orally active EET analogues and sEH inhibitors [11]. Both, EET analogues and sEH inhibitors, showed positive morphological, hemodynamic and mortality effects in animal HF models in our recent studies [12,13]. However, the potential role of eicosanoids in clinical cardiovascular disease particularly in HF, is not fully understood [14,15].

The aim of the study was to investigate the eicosanoids and lipid profiles as a biomarker panel for predicting survival in patients with de novo or acute decompensated chronic HF.

## 2 Materials and methods

### 2.1 Chemicals and reagents

Acetonitrile (ACN), isopropanol (IPA), water, and ammonium acetate (AmAc), all in liquid chromatography-mass spectrometry (LC-MS/MS) grade, were purchased from Sigma-Aldrich (St. Louis, MO, USA). As internal standards, the SPLASH® LIPIDOMIX® Mass Spec Standard mixture and ceramide (d18:1-d7/15:0) were used and purchased from Avanti Polar Lipids (Alabaster, AL, USA). Arachidonic acid-d8 was purchased from the Cayman Chemical Company (Ann Arbor, MI, USA). As the standard reference material for plasma, we used the NIST® SRM® 1950 - “Metabolites in frozen human plasma” (SRM 1950) which was purchased from Sigma-Aldrich (St. Louis, MO, USA).

### 2.2 Patient groups

The study included 50 patients (16 females, aged from 30 to 96 years, mean age 73 years and 34 males, aged from 46 to 86 years, mean age 71 years) with de novo or acute decompensated chronic HF admitted to our tertiary cardiovascular centre. This study was approved by the Ethics Committee of University Hospital Motol (number EK-739/21, approved on 16.6. 2021). Written informed consent was obtained from all participants enrolled in the study. During the prospective median follow-up of 7 months, 13 patients died (hereafter referred to as the non-surviving group). The patients were characterised by cardiological assessments, including the left ventricular ejection fraction (LVEF). Biochemical parameters (CRP, NT-proBNP, LDL, creatinine) were determined using commercially available biochemical and immunochemical assays. Furthermore, eGFR was calculated using the CKD-EPI equation. Clinical data and risk factors were analysed. A summary of the patient's characteristics is provided in Table 1 and provided in detail in Table S1.

**Table 1:** Patient characteristics. Data are presented as median (interquartile range, Q1/Q3) or number (%) and p-values correspond to the two-tailed Mann-Whitney test (continuous variables) or Fisher's exact test (categorical variables).

Description of the patient cohort	Surviving group (n = 37)	Non-surviving group (n = 13)	p-value
Age (years)	76 (68/80)	81 (71/86)	0.0698
Female (%)	37.8	15.4	0.1792
BMI (kg/m^2^)	28 (25.1/31.1)	29.4 (27.4/31.9)	0.3265
Weight (kg)	82 (75/94)	85 (75/90)	0.4395
HFrEF (%)	51.4	76.9	0.1906
HFmrEF (%)	13.5	0	0.3087
HFpEF (%)	35.1	23.1	0.5075
LVEF (median %)	40 (27/55)	25 (20/40)	0.1221
Statin (%)	59.5	46.2	0.5204
Hypertension (%)	81.1	69.2	0.4446
Type 2 diabetes mellitus (%)	48.7	46.2	>0.9999
Coronary artery disease (%)	78.4	69.2	0.7069
COPD (%)	24.3	23.1	>0.9999
Chronic kidney disease (eGFR <1 ml/s/1.73m^2^) (%)	48.7	76.9	0.1084
**Biochemical characteristics**			
NT-proBNP (ng/l)	3785 (2458/8541)	11263 (7185/26269)	**0.0155**
eGFR (ml/s/1.73m^2^)	1.0 (0.7/1.3)	0.5 (0.4/0.6)	**0.0019**
CRP (mg/l)	6.8 (1.9/13.3)	23.5 (17/47.1)	**0.0030**
LDL cholesterol (mmol/l)	2.4 (1.7/3.1)	1.7 (1.3/2.5)	0.1149
Total cholesterol (mmol/l)	4.1 (3.3/4.9)	3.1 (2.9/3.9)	0.0994

Abbreviations: HFrEF - heart failure with reduced ejection fraction (LVEF ≤40 %), HFmrEF - heart failure with mildly reduced ejection fraction (LVEF 41–49 %), HFpEF - heart failure with preserved ejection fraction (LVEF ≥50 %), LVEF - left ventricular ejection fraction, NT-proBNP - N-terminal-prohormone brain natriuretic peptide, COPD - chronic obstructive pulmonary disease, eGFR - estimated glomerular filtration rate, CRP - C-reactive protein, LDL - low-density lipoprotein, SD - standard deviation, BMI - body mass index.

### 2.3 Serum preparation and eicosanoid analysis

Sample collection, preparation and storage were carried out with a consideration to minimize preanalytical effects [16]. Blood samples were collected the next day (after admission) in the morning before breakfast in tubes coated with microscopic silica particles as a coagulation activator. After centrifugation (10 min, 4000 rpm, 8 °C), the serum was separated, aliquoted, directly analysed (eicosanoid analysis) and the rest was stored at −80 °C (for lipidomic analysis). The concentration of eicosanoids (14,15-EET, 14,15-DHET and 20-HETE) was determined using commercially available sandwich ELISA assays (MyBioSource, USA). The ELISA assays were performed according to the manufacturer's instructions and previously published protocol [17], and the semiquantitative data are presented in Table S1.

### 2.4 Lipidomic analysis

#### 2.4.1 Sample preparation for lipidomic analysis

Serum samples for lipidomic analysis were firstly placed from −80 °C to −20 °C overnight and thawed on ice the next day. After freeze-thawing and mixing for 10s on a vortex mixer, the extraction was performed using a protocol described in Ref. [18] by mixing 50 μL of serum with 150 μL of IPA containing internal standards (specified in Table S4). Samples were stored in the freezer (-20 °C) overnight for deproteinization and on the following day, the mixture was centrifuged (10 min, 14 000 g, 4 °C). The supernatant above the protein pellet was transferred (approximately 150 μL) into a glass LC-MS vial. A 10 μL aliquot was taken from each sample and pooled as a quality control (QC) sample. The samples were then immediately subjected to LC-MS analysis. Samples were double randomized, firstly during the sample preparation and secondly with respect to the order of the analytical runs. The QC sample was analysed as every 6th injection and was used for the instrument stability monitoring. Five independently prepared replicates of the SRM 1950 were measured during the analysis.

#### 2.4.2 Liquid chromatography

The method for pseudotargeted lipidomic analysis, using liquid chromatography coupled to mass spectrometry, was used in the same settings as in previous studies [19]. The LC separation was performed by an ExionLC™ system (Sciex, Foster City, CA, USA), the data were acquired using QTRAP® 6500+ mass spectrometer (Sciex, Foster City, CA, USA) and the system was controlled by the Analyst software (version 1.6.2, Sciex, Foster City, CA, USA). For the chromatographic separation, the reversed-phase BEH C8 column (2.1 mm, 100 mm, 1.7 μm, Waters, Milford, MA, U.S.A.) was employed. The mobile phase A was ACN: H_2_O (3:2, v/v), and the mobile phase B was IPA: ACN (9:1, v/v), and both contained 10 mM AmAc. The flow rate was set at 0.35 ml/min and the column temperature was 55 °C. The elution gradient started at 32 % B up to 1.5 min, then increased linearly to 85 % B at 15.5 min, then it increased again to 97 % B at 15.6 min, was held for 2.4 min. The gradient then reached its initial composition of 32 % B at 18.1 min and it was held for 1.9 min for equilibration of the column.

#### 2.4.3 Mass spectrometry

The parameters of the ion source and gasses of the mass spectrometer were set accordingly: ion spray voltage, +4500 V and −4500 V; curtain gas, 40 psi; both ion source gases 1 and 2, 60 and 50 psi respectively, and source temperature, 400 °C. Scheduled multiple reaction monitoring (MRM) with a 2-min window was used for the data acquisition. Positive and negative ionization of compounds was performed in one analysis using the polarity switching function of the used mass spectrometer used. Specific acyl-defining MRM transitions in negative mode were calculated using LipidCreator software [20]. These MRM were added to the method for identification of lipid molecular species (acyl-specific identification) where all adducts and fragment types are provided in detail in Table S2. Due to instrumentation capabilities, it was not possible to perform acyl-specific fragmentations for all lipids (limitation of the number of MRM transitions to achieve sufficient sensitivity), but after the identification of statistically significant lipids, these lipids were further fragmented using a QC sample to elucidate their structural composition (Fig. S1). Declustering potentials and collision energies were optimized using deuterated standards as well as the linearity and other analytical parameters, these have been already provided in detail in our previous work using the same lipidomic approach [21]. Lipid elution curves plotted using the R script [22] (Fig. S2) were used to ensure correct lipid annotation.

### 2.5 Data treatment and statistical analysis

Raw data from the lipidomic analysis were processed in SCIEX OS software (Sciex, version 1.6.1) using semiautomatic peak integration algorithm. Peak areas were divided by areas of their internal standards (always one internal standard per lipid (sub)class, listed in Table S4). The concentration calculated in this way (for which Type I correction was applied) [23] should be considered semiquantitative (level 3) according to the guidelines of the Lipidomics Standards Initiative [24]. The concentrations of lipids are shown in Table S5 and the calculation of the concentration is described in our previously published work [19]. A comparison of the concentration of lipids in SRM NIST 1950 with the reference values is shown in Fig. S3 (and in detail in Table S6). Pareto scaling, and mean centering were applied to the final dataset (Table S3). Statistical evaluation of the data was performed in GraphPad (version 9.0, San Diego, California, USA), SIMCA software (version 15.0, Umetrics, Umeå, Sweden), R program (4.0.3) [25] using the Metabol package [26] and IBM SPSS Statistics (28.0.1.1). Data were analysed using both multivariate (principal component analysis, PCA; orthogonal partial least squares discriminant analysis, OPLS-DA) and univariate (box plots, Mann-Whitney *U* test, fold change) methods. The Cytoscape program (https://cytoscape.org/) was used for global visualization of changes occurring in lipid profiles [27]. In the Cytoscape visualization (Fig. 2), each of the detected compounds was represented by a circle (node). The size of the nodes represented the -log p-value and the colour was based on fold-change (shades of red/blue represented an increase/decrease between two tested groups). The p-value from the Mann-Whitney *U* test was corrected by the Benjamini-Hochberg approach (Table S7) [28]. The ROC analysis and Kaplan-Meier survival analysis were performed to evaluate the diagnostic and predictive power of lipids and eicosanoids in the patient's cohort where a p-value <0.05 was considered statistically significant (Table S7). The raw data and Tables S1–7 have been uploaded to the MassIVE database and are publicly available under the provided link: https://doi.org/10.25345/C5J960F34. To evaluate the associations of selected lipids with biochemical and physiological parameters, we have applied Spearman correlation analysis (where correlations of 0–0.19, 0.20–0.39, 0.40–0.59, 0.60–0.79, and 0.80–1.00 were considered as negligible, weak, moderate, strong and very strong, respectively) (Fig. S4). A Cox regression model was fitted using GraphPad Prism (version 10.0, San Diego, CA, USA) to calculate hazard ratios (HR) associated with HF death occurring during 7 months follow-up using days from admission to death or end of follow-up as the time scale (Table S10).

The power of the study was evaluated and an effect size >0.84 (Cohen's D) was calculated for the comparison of the non-surviving (N = 13) and surviving (N = 37) groups to be statistically significant for the two-tailed *t*-test under the conditions of type I error (alpha = 0.05) and required power (1 – beta = 0.8).

## 3 Results

### 3.1 Serum lipidomics, map of lipid classes and univariate statistics

A total of 736 lipids and 3 eicosanoids were measured and semiquantified in serum using our analytical setup. The following lipid classes were identified (the abbreviation of the lipid class is given in parentheses followed by the number of lipids in this class): cholesteryl esters (CE, 11), ceramides (Cer, 29), diacylglycerols (DG, 20), free fatty acids (FA, 16), dihexosylceramides (Hex2Cer, 5), monohexosylceramides (HexCer, 16), lysophosphatidylcholines and their plasmanyl/plasmenyl variants (LPC, LPC-O, LPC-P 40), lysophosphatidylethanolamines and their plasmanyl variant (LPE, LPE-O, 16), phosphatidylcholines and their plasmanyl/plasmenyl variants (PC, PC-O, PC-P, 157), phosphatidylethanolamines and their plasmanyl/plasmenyl variants (PE, PE-O, PE-P, 132), phosphatidylinositols (PI, 45), phosphatidylserines (PS, 5), sphingomyelins (SM, 89), triacylglycerols (TG, 154), eicosanoids (3 + 2 ratios) and cholesterol. The high number of lipids for some lipid classes (e.g. TG, PC, PE) is due to the fact that both the total sum and specific acyl variants of these lipids were measured in our analysis and were retained in the dataset for further processing in the subsequent statistical analysis.

**Fig. 1:**
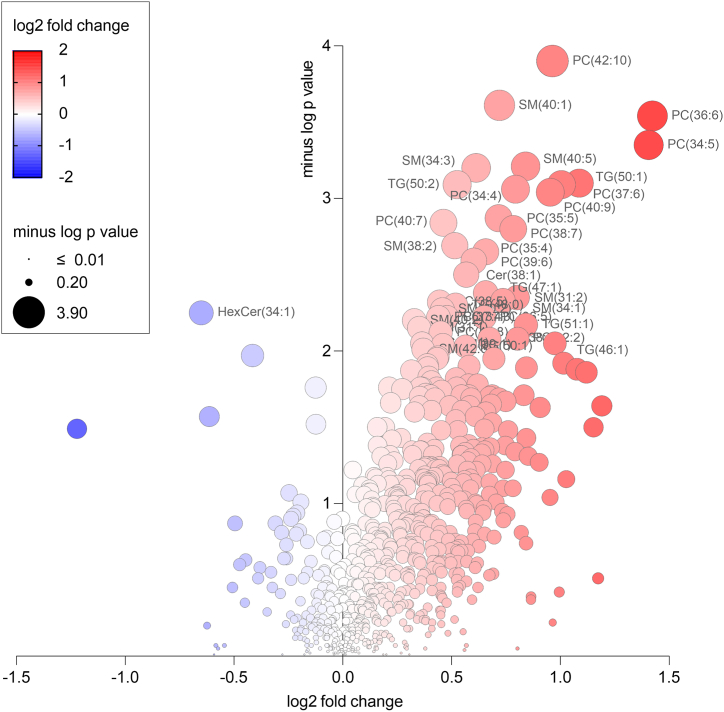
Volcano plot of all 736 lipids. The y-axis and the size of the circles represent the -log p-value (Mann-Whitney *U* test) between surviving vs. non-surviving patients. The colour of the circles and the x-axis represent a log2 fold change of medians between surviving vs. non-surviving patients. Circles with labels correspond to lipids that remained significant after Benjamini-Hochberg correction of the p-value. (For interpretation of the references to colour in this figure legend, the reader is referred to the Web version of this article.)

**Fig. 2:**
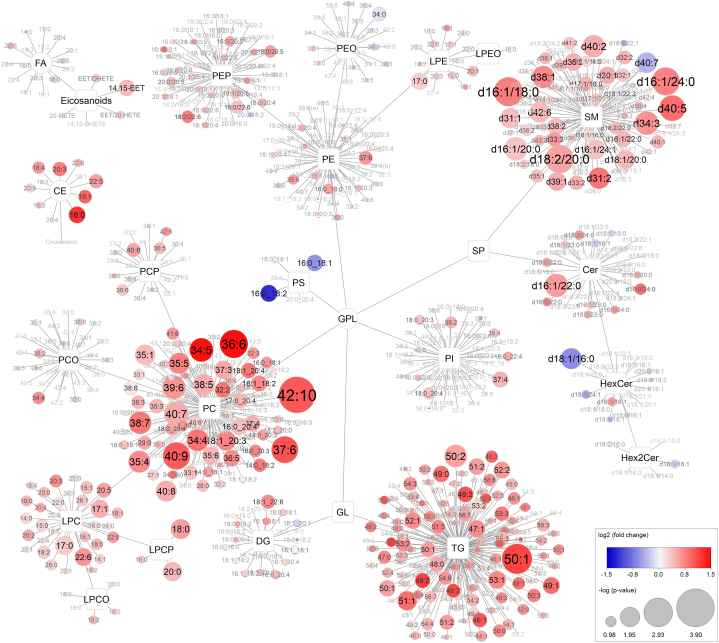
Map of lipids and eicosanoids showing the entire lipidome divided into individual lipid classes and subclasses. The size of the circles represents the -log p-value (Mann-Whitney *U* test) between surviving and non-surviving patients. The colour of the circles represents a log2 fold change of medians between surviving and non-surviving patients. (For interpretation of the references to colour in this figure legend, the reader is referred to the Web version of this article.)

The Mann-Whitney *U* test was used to differentiate between the groups of surviving patients from non-surviving patients during the observation period of our study. After applying the Benjamini-Hochberg correction, 39 altered lipids consisting of 16 PC, 15 SM, 3 TG, 2 LPC-P, LPC, Cer and HexCer were considered statistically significant. A volcano plot was used to visualize a general overview of the changes in lipid concentrations (Fig. 1). The significant PC were mainly polyunsaturated species (more than 5 double bonds on average) such as PC 42:10, PC 36:6, and PC 40:9. Using an additional fragmentation analysis (Fig. S1), to elucidate the structure (acyl-chain composition) of these low abundant long PUFA PC, it was found that PC 42:10, PC 40:9, and PC 40:7 are dominantly represented by PC 20:4_22:6, PC 20:4_20:5, and PC 18:1_22:6, respectively. On the other hand, the significantly increased SM in the surviving group were mono- or diunsaturated (SM d16:1/18:0, SM d18:2/20:0, SM d16:1/24:0), but also polyunsaturated species with 40 and more carbon atoms such as SM d40:5, and SM d42:6. Significant TG contained 50–51 carbons and 1–2 double bonds, namely TG 50:1, TG 50:2 and TG 51:1. A map of all lipids grouped by lipid class was generated to provide details of all identified and semiquantified lipids (Fig. 2). To investigate the associations of individual parameters with the levels of our proposed lipid markers, we performed a Spearman correlation analysis (Fig. S4) of physiological (age, BMI) and biochemical parameters (LDL, total cholesterol, eGFR, CRP), the CVD biomarker NT-proBNP and all significant (mostly PUFA) PC. Correlation analysis revealed moderate to strong positive correlations between all the PUFA PC but only a moderate positive or negative correlations between the above-mentioned parameters and PUFA PC. For example, the most significantly altered lipid PC 42:10 showed a moderate negative correlation with NT-proBNP (−0.56) and CRP (−0.41), a moderate positive correlation with total cholesterol (0.54) and a strong positive correlation with PC 40:9 (0.86) and PC 40:7 (0.71). The average correlation between significant PUFA PC and age and BMI was close to 0 (−0.05 and −0.04, respectively), which is considered a negligible correlation. In addition, we performed a comparative ROC analysis which showed that the significantly altered PUFA PC had a better clinical performance (higher AUC) as classifiers of surviving vs. non-surviving patients compared to NT-proBNP, CRP and eGFR (Fig. S5). To account for the limited sample size of our study, we randomly (using the Excel function “RANDARRAY”) divided the samples into the training set (approximately ⅔ of the samples) and the validation set (approximately ⅓ of the samples). We performed supervised OPLS-DA analysis (Fig. S7 A, B, C, D) and ROC analysis (Fig. S7 E, F) to compare the results of the training and validation sets.

### 3.2 Detailed lipid analysis based on acyl chain lengths and saturation reveals systematic alterations

**Fig. 3:**
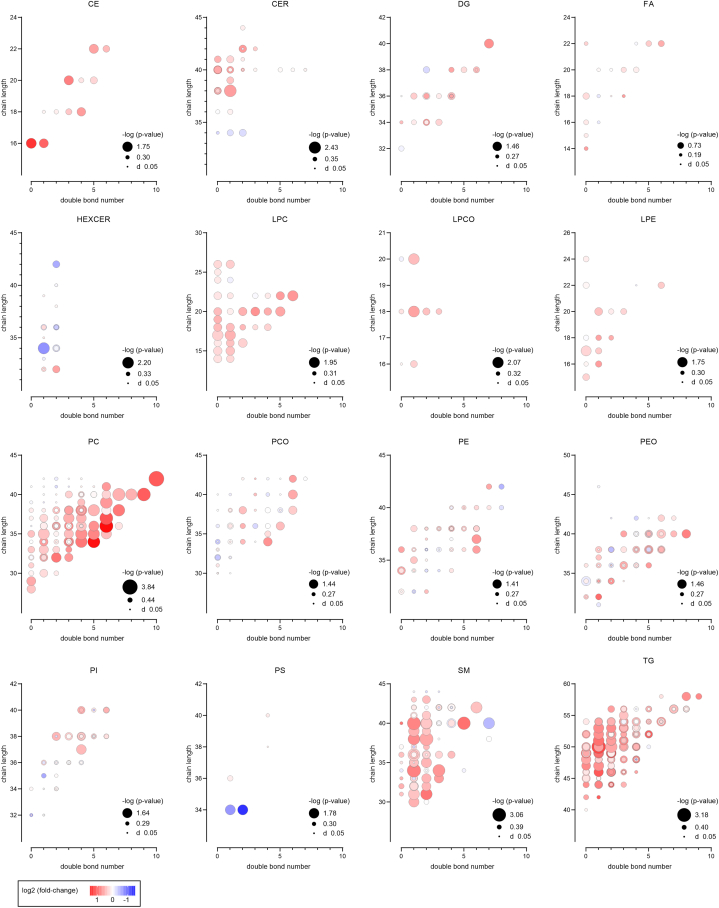
Detailed view into trends of double bonds and acyl chain length of lipids divided into lipid classes and subclasses. The y-axis corresponds to the number of carbons and the x-axis represents the number of double bonds of a total lipid composition. The size of the circles represents the -log p-value (Mann-Whitney *U* test) between surviving and non-surviving patients. The colour of the circles represents the log2 fold change of the medians between surviving and non-surviving patients. (For interpretation of the references to colour in this figure legend, the reader is referred to the Web version of this article.)

To visualize trends in acyl chain length (number of carbons) and number of double bonds, the comparative plots were generated for each lipid (sub)class (Fig. 3). The PC lipid class exhibited the highest number of elevated lipids (in surviving patients) with the highest degree of unsaturation within a given acyl chain length. In the case of LPC, which are biochemically closely related to PC, this trend appears to be less pronounced but still partially present. Conversely, for the SM and TG classes, the most significant changes were observed for mono- and di-unsaturated lipids with longer acyl chains.

### 3.3 ROC and survival analysis based on selected biomarkers

**Fig. 4:**
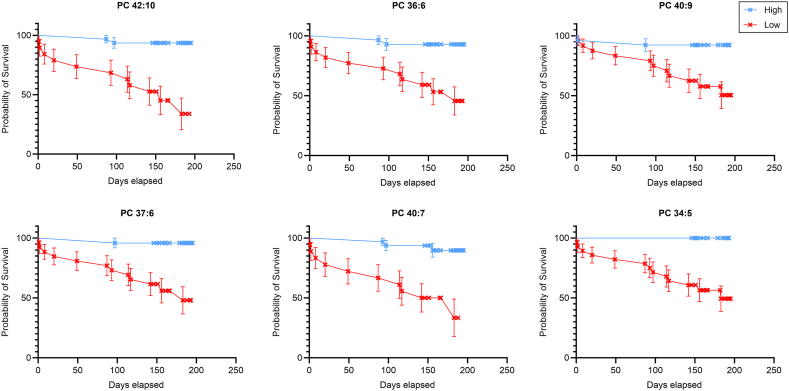
Kaplan-Meier survival curves of the six most significantly decreased PUFA PC in the non-surviving group compared to the surviving group (detailed in Table 2).

The clinical performance of the identified lipid markers was evaluated by ROC analysis (details in Table S7), where a significant result (p-value<0.05) was obtained for 155 lipids and 1 eicosanoid. The ROC analysis of eicosanoids showed a significant prognostic power of 14,15-EET (AUC = 0.72) with respect to the alive-to-death ratio in the patient cohort during the 7-month follow-up study. Similarly, prognostic power was found for multiple lipids across 17 lipid classes listed in Table S8. These lipid classes were (with the number of significant lipids in parentheses): TG (48), PC (37), SM (31), LPC (13), CE (5), LPC-P (2), LPE (2), PC-P (3), PE-P (3), PI (2), PS (2), Cer (2), HexCer (1), DG (1), PC-O (1), PE (1), PE-O (1). The top 10 lipids with the lowest p-value (as determined by ROC analysis) were 6 polyunsaturated PC species with more than 5 double bonds, 3 mono or di-unsaturated SM species and one monounsaturated TG, and their average AUC value was 0.80. The overall average AUC of all significant lipids was 0.71. For each of these lipid classes, one lipid with the lowest p-value of the ROC curve was selected and presented in Table S8. Parameters from the ROC analyses were used to define cut-off values (Table 2 and Table S8), which were subsequently utilised to construct Kaplan-Meier survival curves (Fig. 4 and Fig. S6). We further focused on the systematically altered lipids from the PC class (selected as those with the significant change according to the Mann-Whitney *U* test), specifically PC 42:10, PC 36:6, PC 40:9, PC 37:6, PC 40:7 and PC 34:5, which were used to construct ROC curves (Table 2) and Kaplan-Meier survival curves (Fig. 4, and additionally the log-rank value and p-value in Table S9).

**Table 2:** Results of the ROC analysis of the six most significantly altered PUFA PC showing AUC, Gini index, Max K-S and cut-off values.

Lipid	AUC	Std. Error	Asymptotic significance (p-value)	Gini Index	Max K-S	Cut-off (nmol/ml)
PC 42:10	0.84	0.07	3.235E-07	0.68	0.63	0.19
PC 36:6	0.80	0.06	2.183E-06	0.60	0.58	0.35
PC 40:9	0.79	0.07	2.072E-05	0.58	0.49	0.04
PC 37:6	0.79	0.07	1.633E-05	0.58	0.54	0.72
PC 40:7	0.78	0.06	2.094E-05	0.55	0.55	6.21
PC 34:5	0.77	0.06	2.795E-05	0.54	0.59	0.23

The patients were closely monitored by the clinic and data on exact survival time in days were available for statistical analysis. These data were used to construct Kaplan-Meier survival curves using the cut-off estimates from the ROC analyses (shown in Table 2 and detailed in Table S7). The representative Kaplan-Meier survival curves for the most significantly altered PUFA PC (as listed in Table 2) are shown in Fig. 4.

### 3.4 Multivariate discriminant analysis predicts survival of CVD patients

**Fig. 5:**
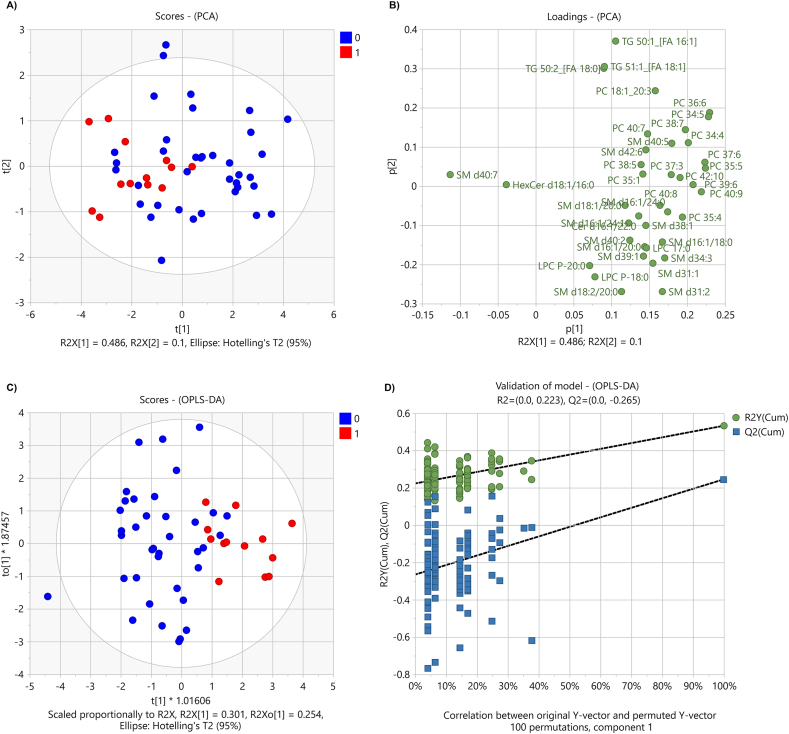
Multivariate statistical analysis. The score plot (A) and loading plot (B) represent the results of an unsupervised principal component analysis. Discriminant analysis (OPLS-DA) score plot (C) and permutation analysis (D) are based on the lipids selected by p-value below the BH-corrected critical value (39 lipids). The red colour in (A) and (C) represents the non-surviving group and the blue colour represents the surviving group of patients. (For interpretation of the references to colour in this figure legend, the reader is referred to the Web version of this article.)

To verify how the defined lipid panel (39 significant lipids, selected by Mann-Whitney *U* test after BH correction) discriminates the group of surviving from non-surviving patients, the data were subjected to unsupervised and supervised multivariate analyses (Fig. 5). A partial grouping of surviving and non-surviving patients (48.6 % explained variance on the first component) was observed in the unsupervised principal component analysis (PCA). The contributions of lipids to the construction of the PCA score plot are shown in the loading plot (Fig. 5, B). Furthermore, supervised orthogonal partial least squares discriminant analysis (OPLS-DA) showed an almost complete separation of the studied groups (Fig. 5, C). Although the OPLS-DA model R2Y value of 0.535 and Q2 value of 0.245 is not considered to be a model with good predictability (threshold >0.5 for both values), the permutation test (Fig. 5, D) showed that the original model yielded higher R2Y and Q2 values than the permutated models. The results of these analyses provide evidence that the combination of selected lipid markers is capable of discriminating between surviving and non-surviving patients. The model achieved an accuracy of 95 % and 77 % was achieved for survival and non-survival patients, respectively (Table 3). The OPLS-DA model correctly classified 35 out of 37 surviving patients and 10 out of 13 non-surviving patients.

**Table 3:** Classification performance of the supervised statistical model (OPLS-DA) from Fig. 5C.

	n	Correct	0	1
**Surviving group (0)**	37	94.59 %	35	2
**Non-surviving group (1)**	13	76.92 %	3	10
Total	50	90.00 %	38	12
Fisher's prob.	1.6e-06			

### 3.5 Validation of results and analysis of covariates

In order to validate the results, the samples were divided into the training set (approximately two-thirds of the samples) and the validation set (approximately one-third of the samples). The results demonstrated that both the training and validation sets showed complete separation of samples from the surviving and non-surviving groups in the OPLS-DA (Fig. S7 A, B). Furthermore, both the training and validation OPLS-DA models demonstrated the ability to correctly classify samples from these two groups with 96.88 % and 100 % model accuracy, respectively (Fig. S7 C, D). Finally, an ROC analysis was performed to compare the clinical performance of the first six most significantly altered PUFA PC which showed, that PUFA PC as markers were able to discriminate between surviving and non-surviving patients in both the training and validation sets, with a mean AUC of 0.76 and 0.83, respectively (Fig. S7 E, F). A Cox regression analysis was conducted to assess whether serum CRP, NT-proBNP, eGFR and PC 42:10 were associated with death after HF. The PC 42:10 was identified as the only significant covariate (HR: 0.058, 95 % CI: 0.008–0.367; p = 0.003), while no significant associations were observed for the remaining covariates (Table S10).

## 4 Discussion

The present study was conducted on patients with de novo or acute decompensated chronic HF who were accepted and treated at our tertiary cardiovascular centre. The study focused on a cohort of patients from a purely clinical setting. Furthermore, this study did not focus on the prediction of events or death in the general population or in those at risk of developing the disease, but rather on patients at an advanced stage of the disease (de novo or acute decompensated HF). The potential of lipids and eicosanoids to distinguish between the surviving and non-surviving groups of patients was evaluated. Our findings indicate that peripheral levels of lipids (mainly PUFA PC) and eicosanoids (especially 14,15-EET) offer significant prognostic power with respect to the alive-to-death ratio in HF patients during 7-month follow-up. These findings suggest that it is possible to stratify this susceptible group from those with a higher chance of survival using our lipid panel.

In terms of lipidomics, we identified numerous significant lipids across lipid classes and subclasses, which belonged primarily to PC, SM, TG, LPC-P, LPC, Cer, and HexCer. Significantly altered lipids (n = 39) were selected using univariate statistics to construct multivariate models. The classification performance of the selected lipids was evaluated using a validated supervised OPLS-DA model which demonstrated an overall 90 % accuracy in classifying patients at risk of death. To provide a more detailed description of the common structural features of these significant lipids, we constructed plots according to the number of carbons and double bonds for each lipid by lipid (sub)class. A clear trend was identified in the PC class, namely that as the number of double bonds increases (towards polyunsaturated species), the significance and clinical performance to distinguish the surviving group from the non-surviving group of patients increases regardless of the number of carbons. Although several individual polyunsaturated PC species such as PC 36:6 or PC 34:4 have been identified as significant both in our and also previous studies [29], our study highlights a yet undescribed systematic phenomenon across this whole lipid class. Upon closer examination, we also found significant changes in long (>40 carbons) and highly polyunsaturated (>7 double bonds) phosphatidylcholines such as PC 40:9, PC 40:7 and especially PC 42:10, which was the most significantly elevated lipid in the surviving patients. Some of these low-abundant long-chain PUFA PC have not yet been identified and even detected in previous studies (due to their unexpected occurrence in plasma) and consequently they are not included in predictive models such as CERT2 and others [6]. Furthermore, the results from the correlation analysis indicated that PUFA PC are moderately correlated with CVD markers associated with cardiac complications and prognosis such as NT-proBNP and CRP, however, in comparison, PUFA PC show better clinical performance as classifiers of non-surviving patients after a heart attack and that PUFA PC are not correlated with BMI nor age. On the basis of the Cox regression analysis, the common HF-related biochemical parameters (NT-proBNP, CRP and eGFR) were not significantly associated with the risk of death after HF. PC 42:10 as the most discriminating PUFA PC (based on univariate statistical analysis) was significantly associated with death after HF, independent of the aforementioned parameters.

These findings are consistent with several studies showing a cardioprotective effect of PUFA (especially linoleic acid) in direct association with all-cause mortality [30]. Many studies are based on the analysis of total free and total esterified fatty acids (usually determined by GC-MS). It has been shown that esterified PUFA in serum are even more strongly associated with CVD mortality than free dietary PUFA [30]. However, GC-MS analysis of esterified fatty acids is performed in a destructive manner, i.e. we only observe the sum of the fatty acids but do not know their origin (which lipids they are derived from). Our work takes this knowledge a step further by measuring the individual lipids in which the fatty acids are esterified thus allowing us to identify which lipid class is most important in relation to the cardioprotective function of esterified PUFA during HF. The structural composition of these low abundant lipids at the molecular species level was confirmed by fragmentation experiments. According to our results, the PUFA with the most significant cardioprotective role with respect to survival after HF are mainly FA 22:6, FA 20:4 and FA 20:5 esterified in PC.

Moreover, myocardial phospholipid remodelling has been observed in association with various forms of heart failure, often manifesting as lower levels of linoleic acid and a reciprocal elevation of long-chain unsaturated fatty acids, such as FA 22:6 and FA 20:4 [31,32]. In contrast to the myocardial tissue, in plasma and serum, the levels of free and esterified PUFA (mainly ω-3 species) are elevated in both patients with low CVD risk and lower risk of CAD [6,33,34]. This observation may be related to the increased activity of δ-6 desaturase (D6D), the rate-limiting enzyme in PUFA biosynthesis [32]. Although D6D inhibition reversed the pathological manifestation of HF in an animal model [32], in our study elevated serum PUFA esterified in PC are associated with surviving HF patients. Several studies have investigated the beneficial effects of PUFA on cardiac function. Omega-3 FA have been shown to suppress the expression of pro-inflammatory cytokines and the infiltration of inflammatory cells into the heart. In subjects with stable ischemic heart failure), supplementation with omega-3 PUFAs has been shown to improve endothelial function, inflammatory and fibrotic status [35,36]. Further research is needed to elucidate the role of PUFAs in the pathobiochemistry of HF.

In addition, particular attention was paid to eicosanoids. The reduced availability of biologically active epoxygenase products expressed as the EET/DHET ratio is commonly used to assess the bioavailability of these metabolites [[37], [38], [39]] as was also validated in our preclinical study, where this ratio was reduced by approximately 65 % in the kidney and left ventricle (LV) of the animal CHF model as compared to controls [12,13]. There were no significant differences in the protein expression of the enzymes responsible for EETs production in the kidney and LV. However, the expression of sEH protein, an enzyme responsible for converting EETs to DHETs, was significantly increased in the animal HF model in LV tissue [13]. In this human pilot study, we did not measure the direct tissue concentrations of eicosanoids in the heart or kidney, where we would expect a deficiency of active EETs based on our previous preclinical data. In addition, we investigated the serum levels of 14,15-EET, 14,15-DHET and 20-HETE in our patient cohort. We demonstrated significant diagnostic power of 14,15-EET in patients with a significant cut-off of 0.24 nmol/mL (78.18 ng/mL).

The increased peripheral concentration of eicosanoids may be a compensatory mechanism for decreased availability in cardiac and renal tissues and to counterbalance the activated renin-angiotensin system [[40], [41], [42]]. Several body compartments contribute to peripheral plasma levels of eicosanoids, as different cell types produce eicosanoids that act in a paracrine and autocrine manner [40]. Low peripheral eicosanoid levels may indicate more advanced heart failure with a worse prognosis [38,39].

Our study has several limitations. First, we enrolled de novo or acutely decompensated chronic HF patients because they are at higher risk of CVD events and lipidomics including eicosanoid levels of stable HF patients remain to be elucidated in the next part of our project. Second, the study size is relatively small, with a median age of 74 years and therefore follow-up is short (median of 7 months), thus our exploratory results should be validated in a broader spectrum of HF patients with bigger cohorts and longer follow-up. As our work is a pilot study with a limited number of samples our results should be validated on a larger set of samples where an extended analysis of confounding factors should be performed and evaluated using logistic regression and Cox proportional hazard models. Additionally, a more complex and sensitive analysis of eicosanoids (and possibly also other oxylipins) using LC-MS may provide a deeper insight into the pathobiochemical mechanism of heart failure and risk of death and should be the focus of further studies.

## 5 Conclusions

Lipidomics is an important tool for predicting complications in CVD and HF. The results of our lipidomic and eicosanoid analysis show that the largest changes related to survival after HF occur at the level of long-chain polyunsaturated PC (namely PC 42:10, PC 40:9 and PC 36:6), where a strong systematic trend is observed. A further focus should be placed on low-abundant lipid markers, which have been omitted in many publications and predictive models.

## CRediT authorship contribution statement

**Aleš Kvasnička:** Writing – original draft, Visualization, Validation, Software, Methodology, Investigation, Formal analysis, Data curation. **Karel Kotaška:** Writing – review & editing, Visualization, Supervision, Resources, Project administration, Investigation, Funding acquisition, Formal analysis, Data curation, Conceptualization. **David Friedecký:** Writing – original draft, Visualization, Validation, Supervision, Software, Resources, Project administration, Methodology, Investigation, Funding acquisition, Formal analysis, Data curation, Conceptualization. **Karolína Ježdíková:** Writing – review & editing, Resources, Methodology, Investigation, Conceptualization. **Radana Brumarová:** Writing – review & editing, Methodology, Investigation. **Tomáš Hnát:** Writing – review & editing, Resources, Methodology, Investigation. **Petr Kala:** Writing – review & editing, Visualization, Resources, Project administration, Methodology, Investigation, Funding acquisition, Formal analysis, Data curation, Conceptualization.

## Ethics and consent

This study was approved by the Ethics Committee of the University Hospital Motol (number EK-739/21, approved on 16.6. 2021). Written informed consent was obtained from all participants enrolled in the study.

## Data availability statement

The datasets generated and/or analysed during the current study including all the raw data, additional files (containing Figs. S1–S7 and Tables S1–S10) are available in the MassIVE repository, https://doi.org/10.25345/C5J960F34.

## Declaration of competing interest

The authors declare that they have no known competing financial interests or personal relationships that could have appeared to influence the work reported in this paper.
