# Supplementary material for: Long-chain polyunsaturated fatty acid-containing phosphatidylcholines predict survival rate in patients after heart failure
Source: Heliyon. 2024 Oct 30;10(21):e39979. doi: 10.1016/j.heliyon.2024.e39979 (PMC11567051; doi:10.1016/j.heliyon.2024.e39979)
Supplement: Multimedia component 1 [file mmc1.docx]

**SUPPLEMENTARY INFORMATION**

**Long-chain polyunsaturated fatty acid-containing phosphatidylcholines predict survival rate in patients after heart failure**

Aleš Kvasnička ^a,b,1^, Karel Kotaška ^c,1^, David Friedecký ^a,b,*^, Karolína Ježdíková^c^, Radana Brumarová^b^, Tomáš Hnát ^d^, Petr Kala ^d,e*^

^a^ Laboratory for Inherited Metabolic Disorders, Department of Clinical Biochemistry, University Hospital Olomouc, Czech Republic

^b^ Faculty of Medicine and Dentistry, Palacký University in Olomouc, Czech Republic

^c^ Department of Medical Chemistry and Clinical Biochemistry, 2nd medical faculty, University Hospital Motol, Prague, Czech Republic

^d^ Department of Cardiology, University Hospital Motol and 2^nd^ Faculty of Medicine, Charles University, V Úvalu 84, 150 00, Prague, Czech Republic

^e^ Center of Experimental Medicine, Institute of Clinical and Experimental Medicine, Vídeňská 1958, 140 21, Prague, Czech Republic

^1^ These authors contributed equally to this work and should be considered shared first authors.

* Corresponding authors

**Number of supplementary figures:** 7

**Number of supplementary tables:** 10

**Fig. S1** Extracted ion chromatograms of the most significantly altered long-chain polyunsaturated fatty acid‑containing phosphatidylcholines: PC 42:10 (A), PC 40:9 (B) and PC 40:7 (C). The upper panel shows the quantification multiple-reaction monitoring transition (MRM) in the positive mode, and the lower panel shows the precursor and acyl fragments [FA-H]^-^ MRM for different possible combinations of acyls in the negative mode. Each MRM is color-coded to correspond with the label where m/z values are provided as Q1/Q3.

**
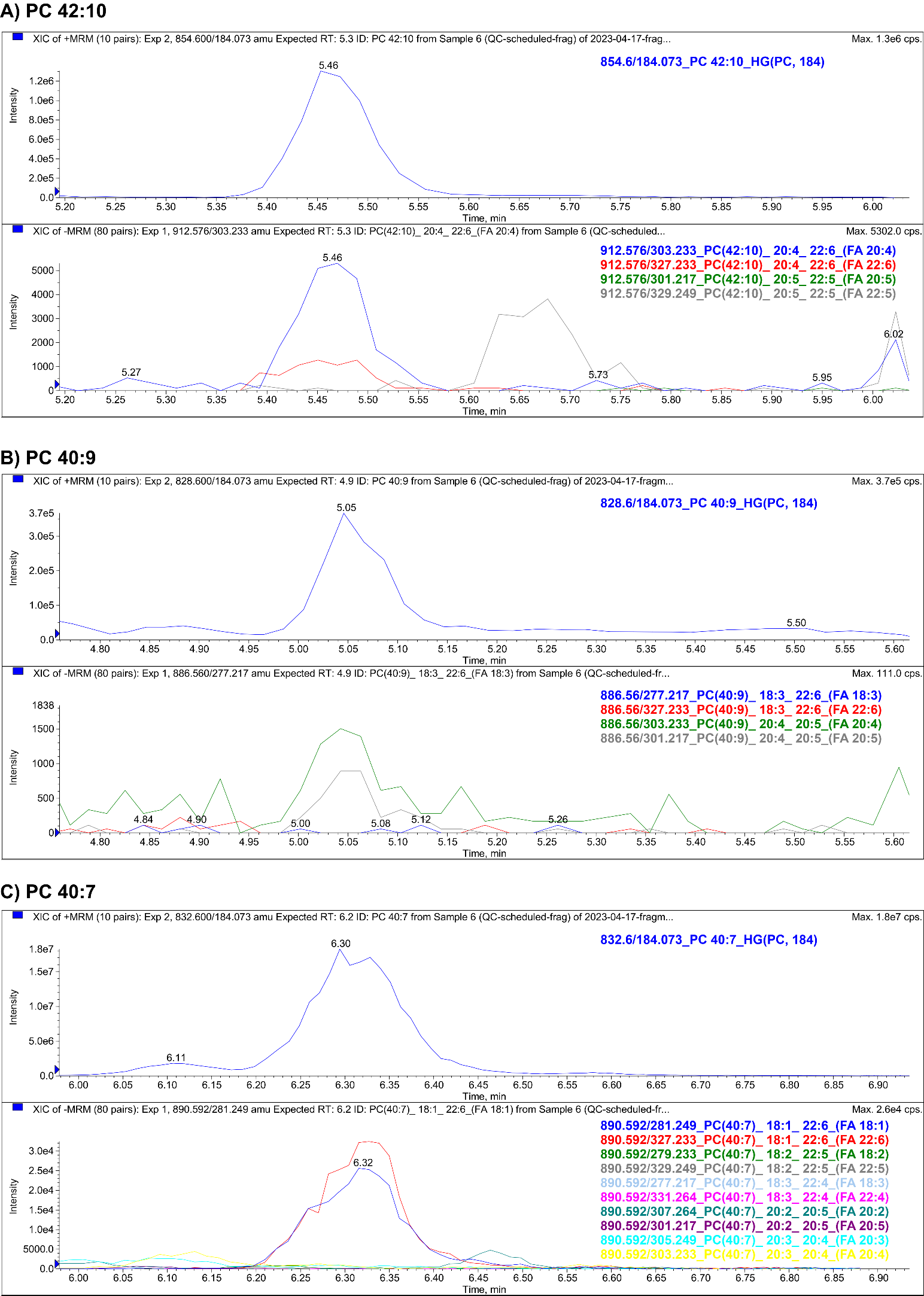
**

**Fig. S2** Lipid patterns plotted for each lipid class. Charts are plotted as *m/z* value on the y-axis and retention time in minutes on the x-axis. Saturation equals to the number of double bonds. Lipid pattern plots were used to correctly assign lipid annotations and to annulate potential misidentifications. Few shifts can be observed for example in ceramides with 1 double bond, which is occurring due to different long-chain base composition (d16:1, d18:1, or d20:1), or in the case of PC-O species where both ether (plasmanyl) and vinyl ether (plasmenyl) species are considered in one plot. Lipid isomers with different acyl-composition can also differ in retention time resulting in non-absolute linear/quadratic pattern curve observed (for example in the case of PC 36:4).


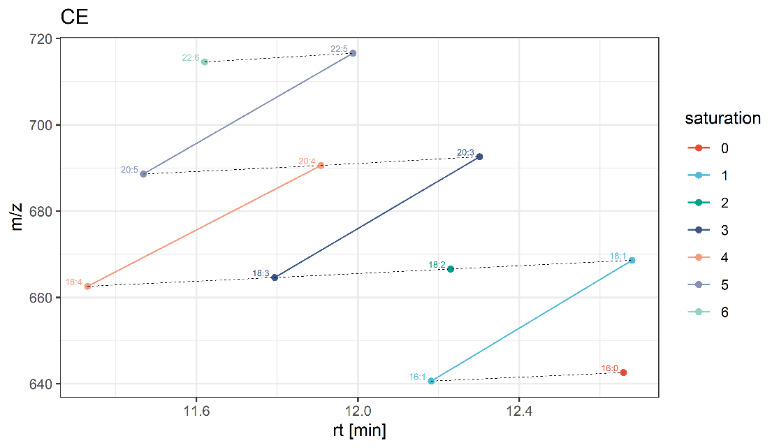

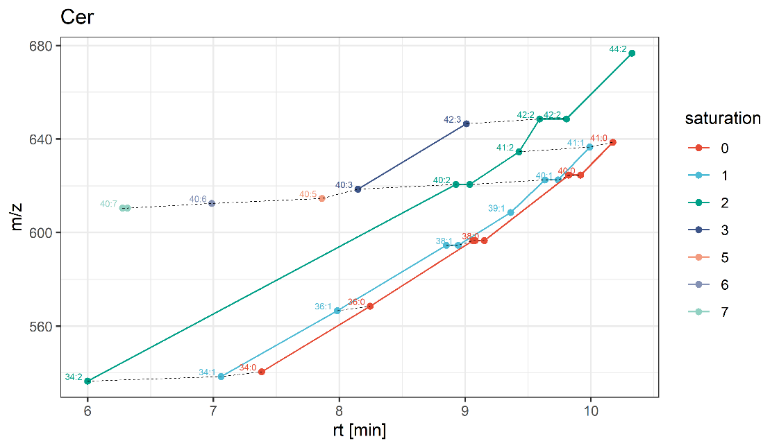

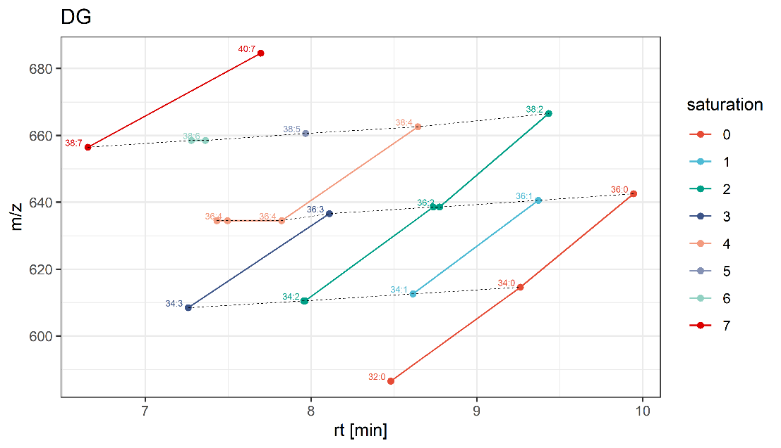

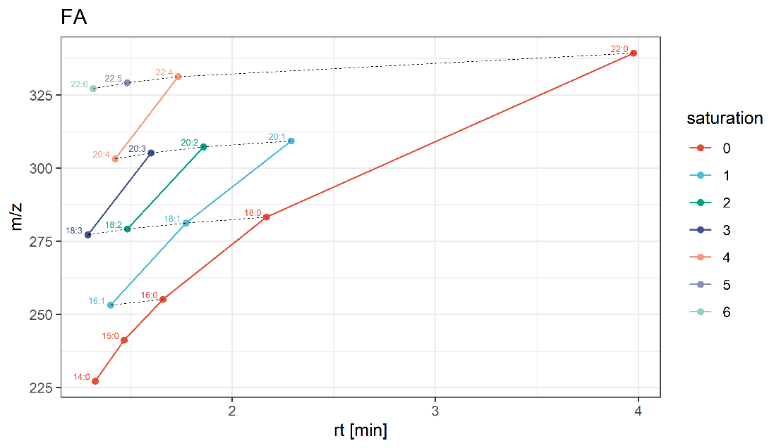

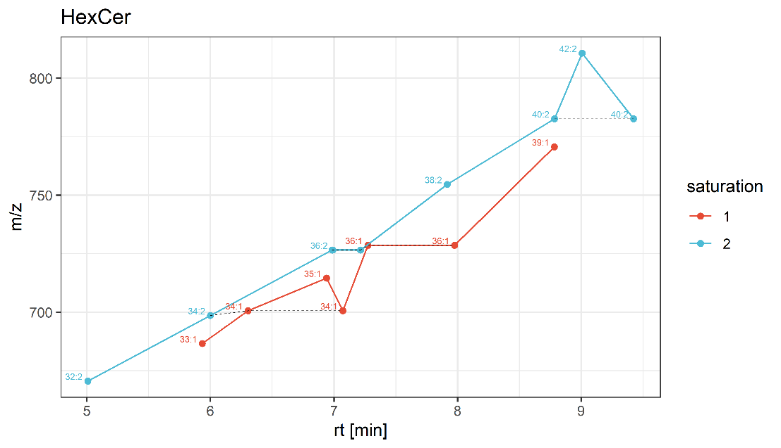

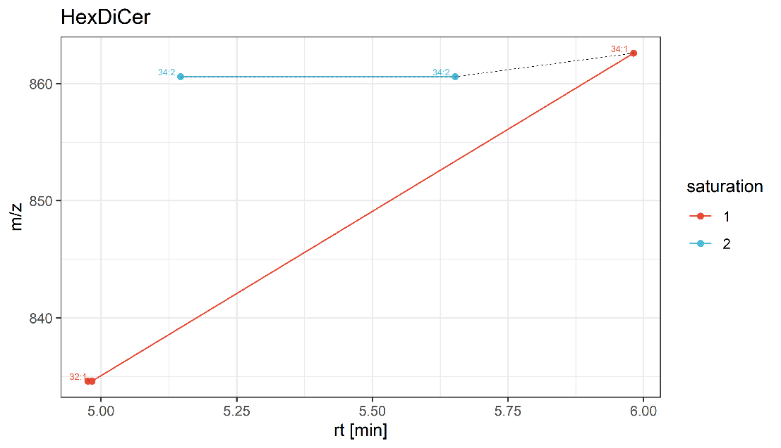

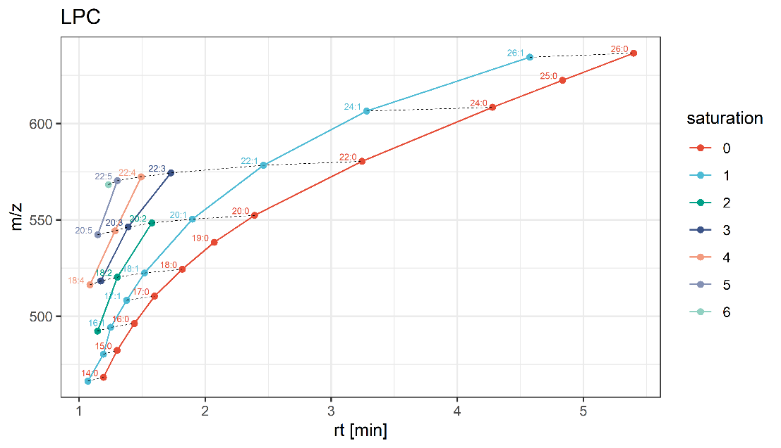

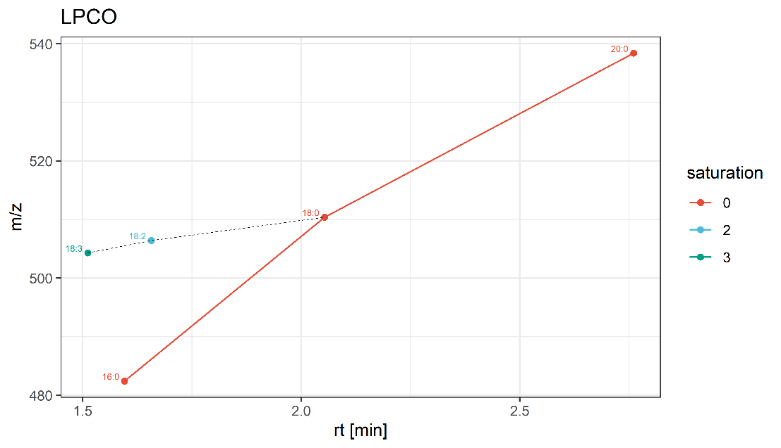

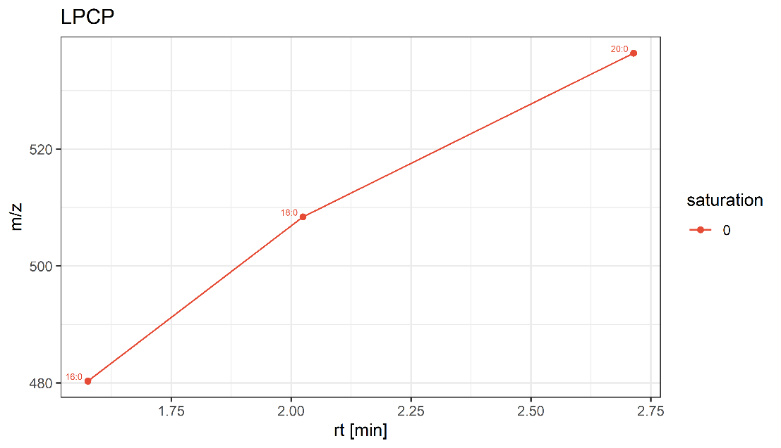

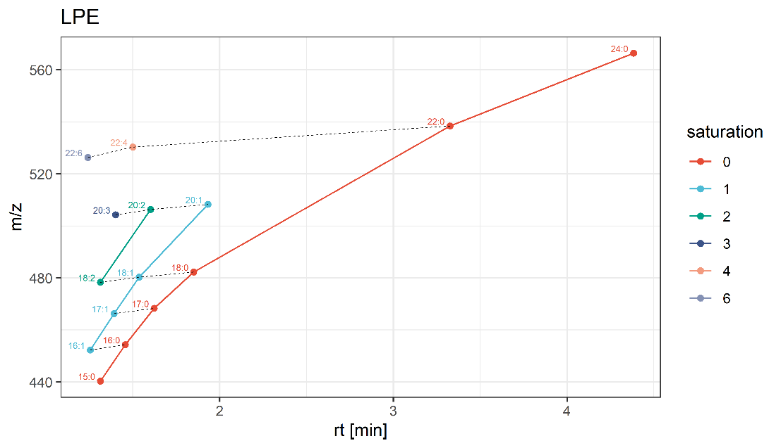

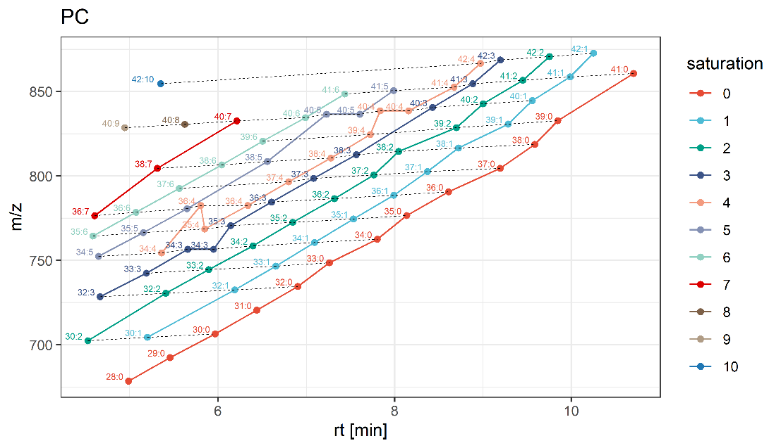

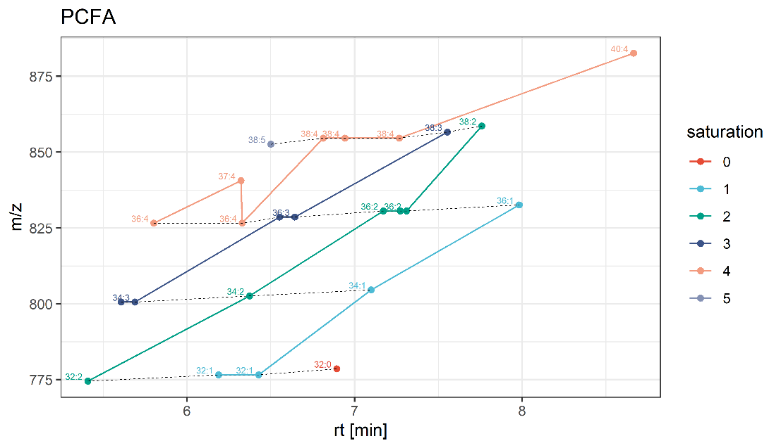

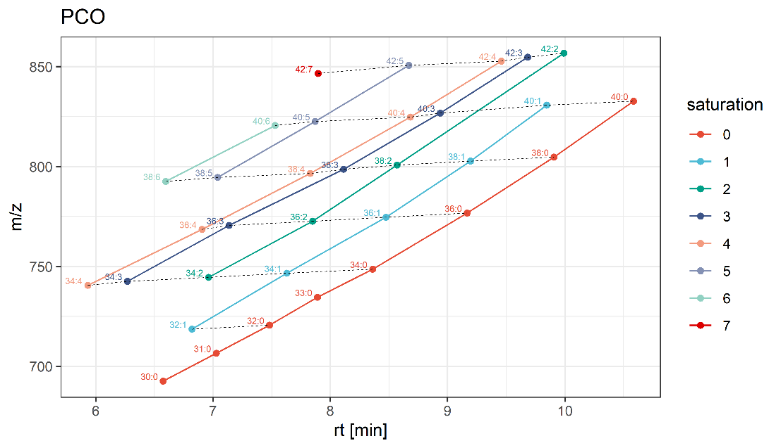

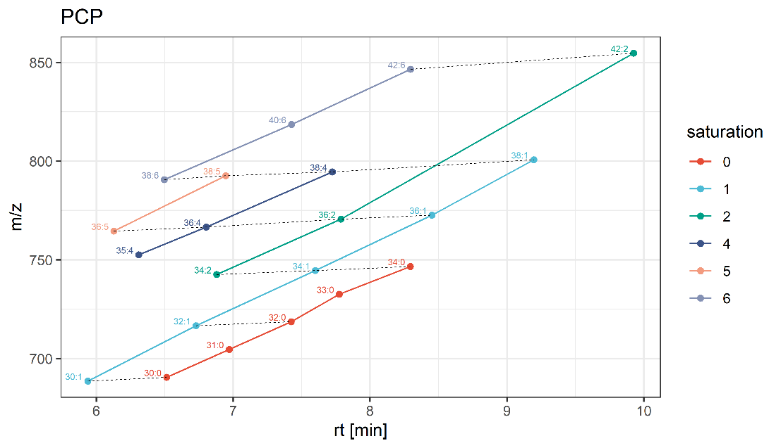

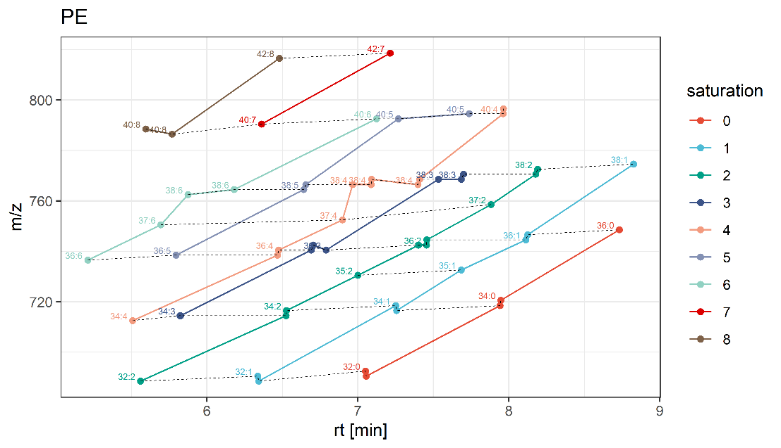

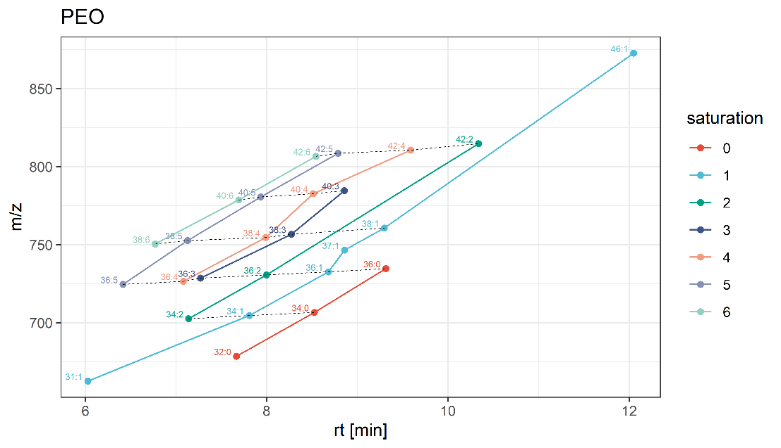

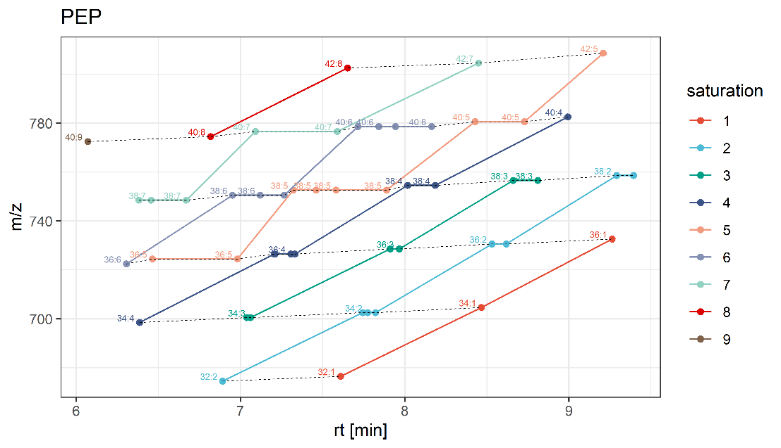

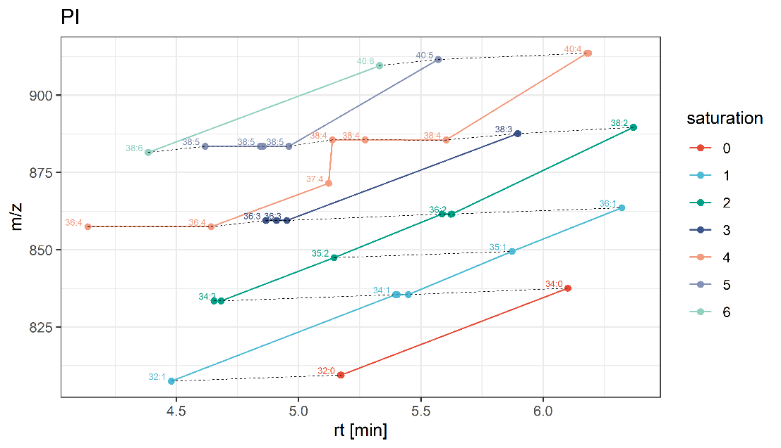

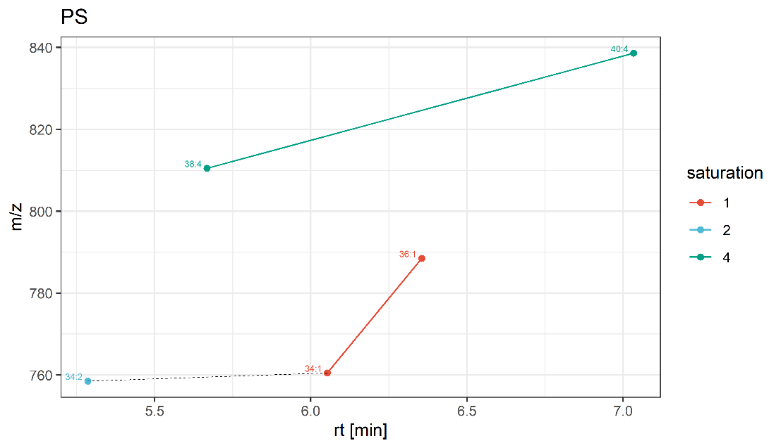

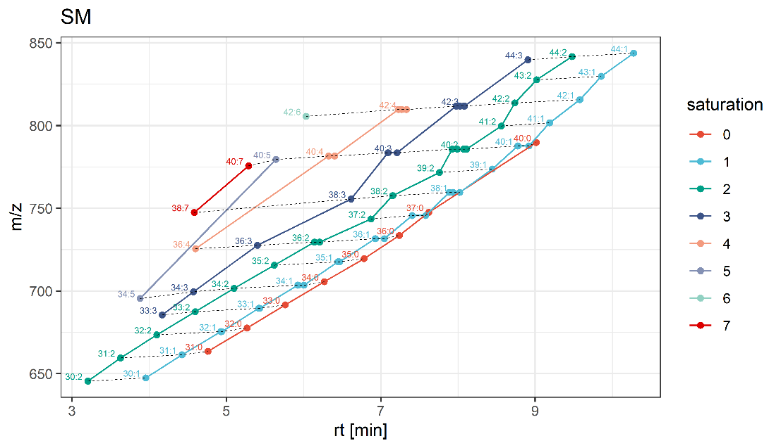

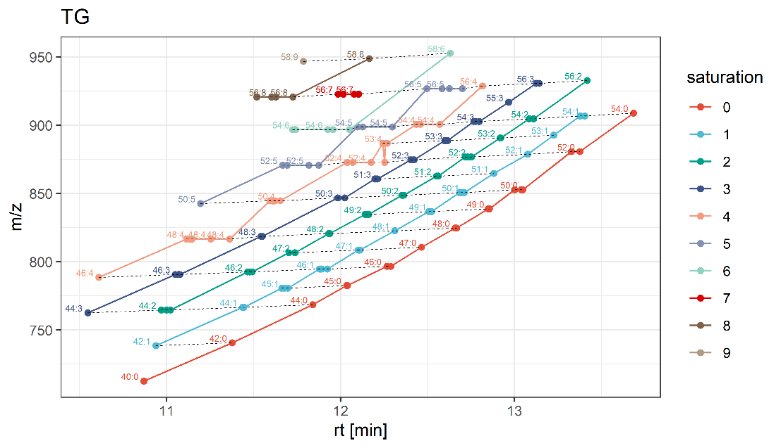


**Fig. S3** Accuracy assessment for SRM 1950 - "Metabolites in Frozen Human Plasma" (number of independently prepared replicates n=5). Values are presented as normalized coverage equivalents at the mean (dots) and stdev (error bars) of measurements, overlaid onto the consensus mean value (blue line) and uncertainty (95% coverage-green region, 99% coverage-red region).

Cholesteryl esters (CE) and free cholesterol Ceramides (Cer)

Diacylglycerols (DAG) Free fatty acids (FFA)

Hexosylceramides (HexCer, Hex2Cer) Lysophosphatidylethanolamines (LPE, LPE O-)

Lysophosphatidylcholines (LPC, LPC O-, LPC P-)

Phopshatidylcholines (PC, PC O-, PC P-)

Phosphatidylethanolamines (PE, PE O-, PE P-)

Phosphatidylinositols (PI)

Sphingomyelins (SM)

Triacylglycerols (TAG)

**Fig. S4** Spearman correlation heatmap for selected physiological, biochemical, and other parameters and most significantly altered lipids from the PC class. Correlations of 0-0.19, 0.20-0.39, 0.40-0.59, 0.60-0.79, and 0.80-1.00 are considered as negligible, weak, moderate, strong, and very strong, respectively.

**
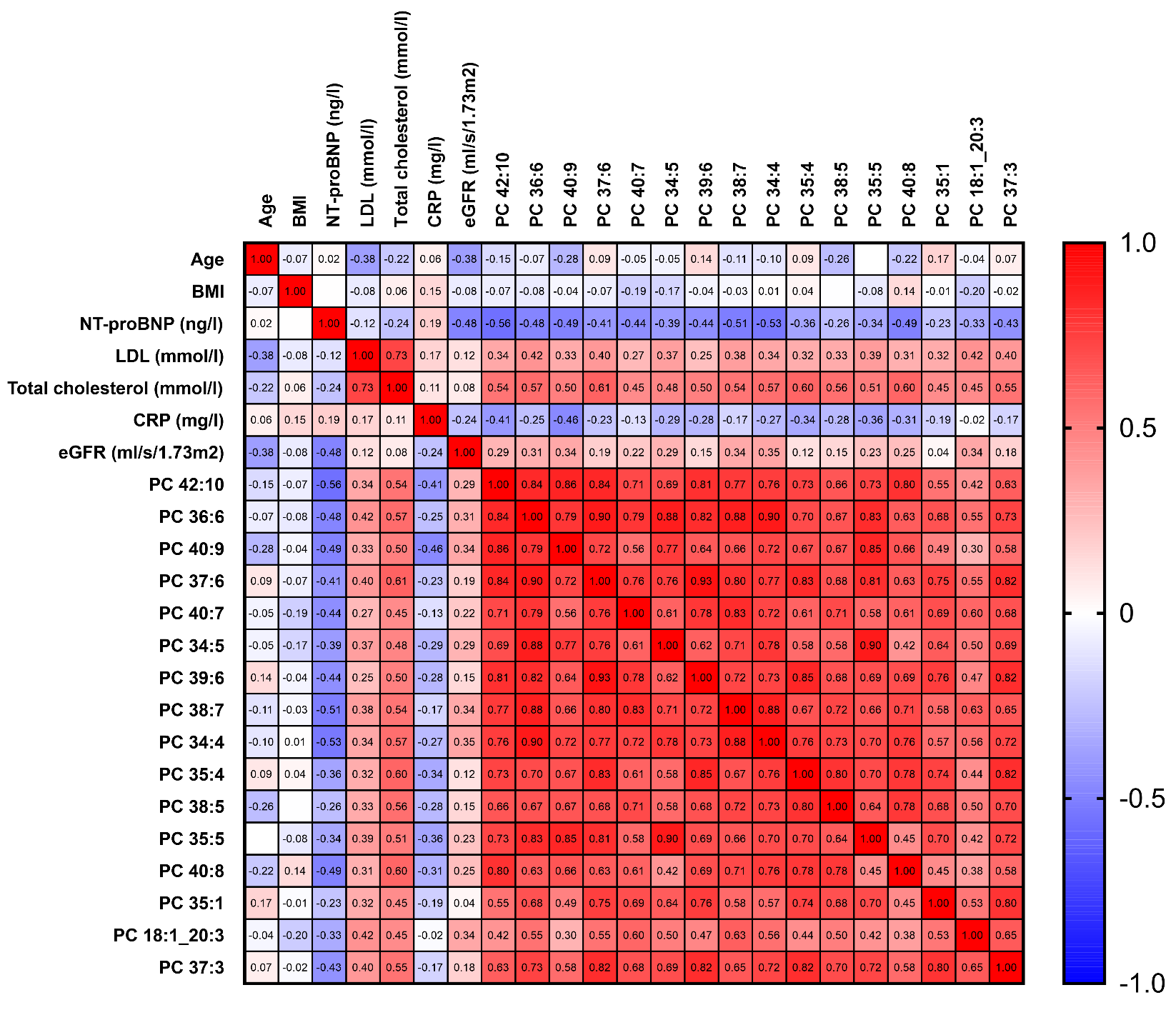
**

**Fig. S5** ROC analysis of selected biochemical parameters (A) and six most significantly altered lipids from the PC class (B).

**
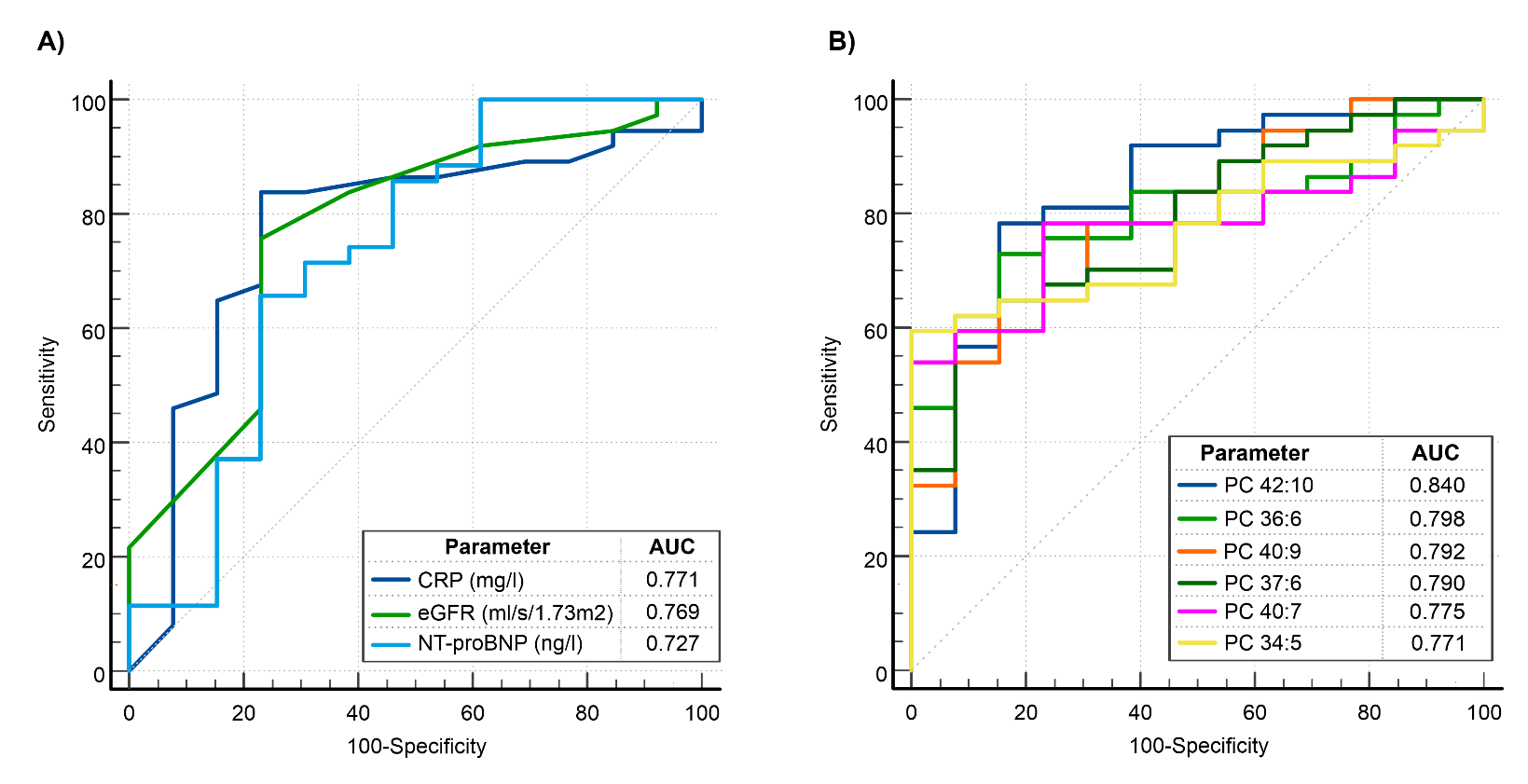
**

**Fig. S6** Kaplan-Meier survival curves of the six most significantly decreased PUFA PC in the non-surviving group compared to the surviving group (specified in Table S8).


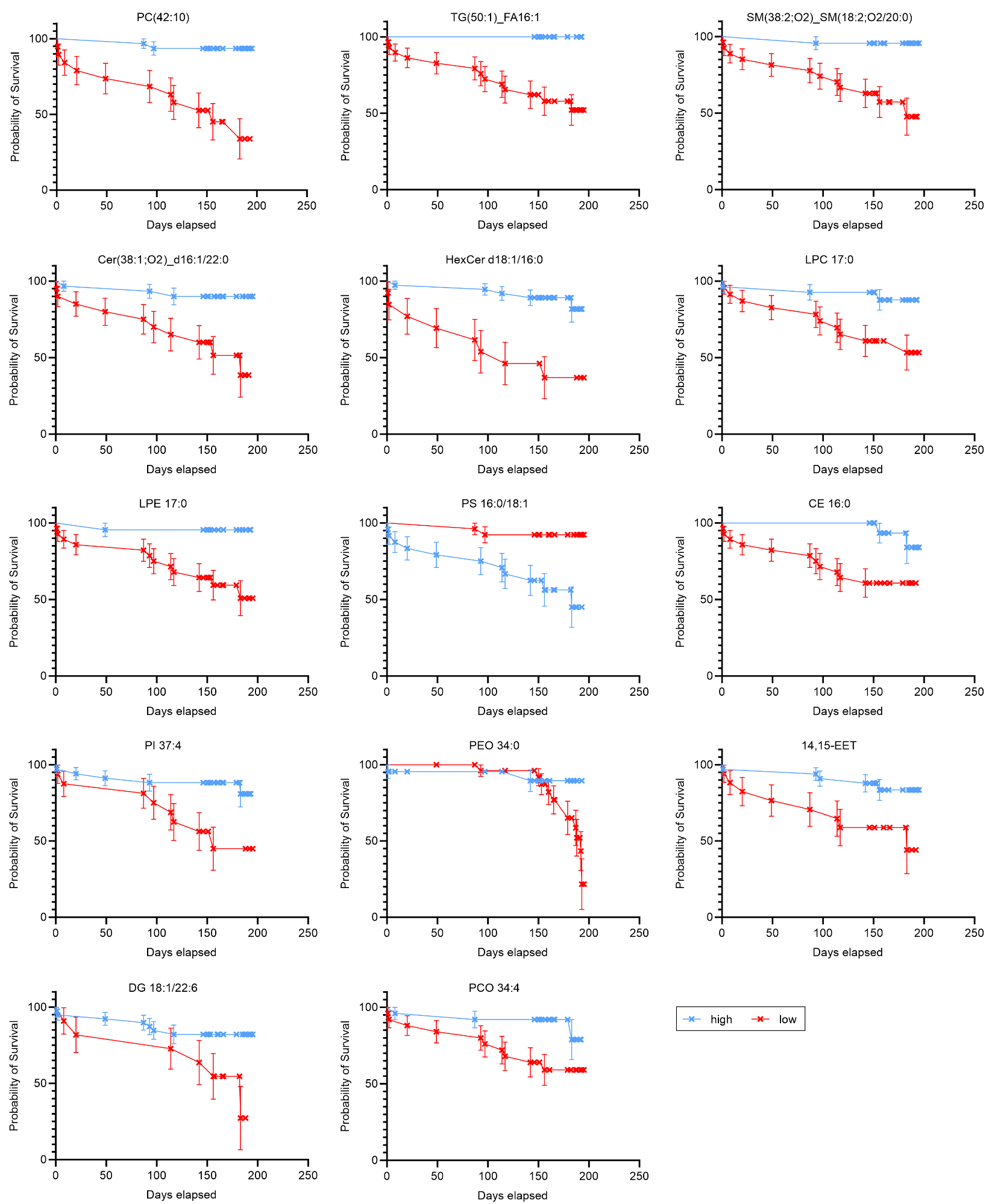


**Fig. S7** Evaluation from the results validation**.** The samples were divided to training set (2/3 of samples) and validation set (1/3 of samples). These sets were separately statistically analyzed by OPLS-DA (A and B), by the classification capabilities of the OPLS-DA model (C and D) and ROC analysis (E and F).

**
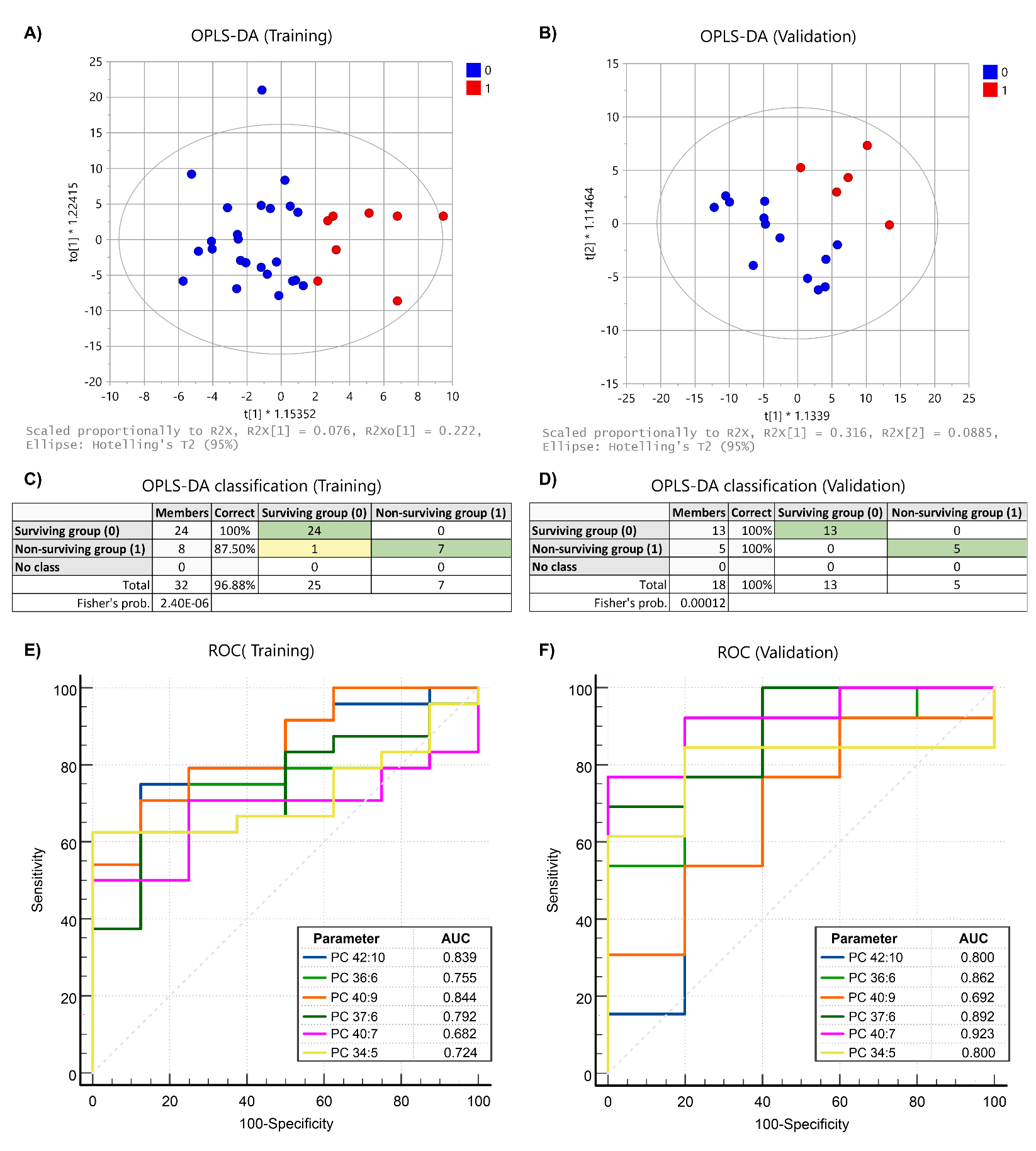
**

**Table S1.** Clinical metadata about patients and determinated levels of eicosanoids (in ng/ml).

| **ID** | **Name** | **Date of admission and sampling** | **Death during follow-up period (1-yes, 0-no)** | **Date of death (or end of follow-up period)** | **Days of survival** | **NT-proBNP (ng/l)** | **LDL (mmol/l)** | **Total cholesterol (mmol/l)** | **CRP (mg/l)** | **eGFR (ml/s/1.73m2)** | **14,15-EET (ng/mL)** | **20-HETE (ng/mL)** | **14,15-DHETE (ng/mL)** | **EET/DHETE** | **EET/20-HETE** |
| --- | --- | --- | --- | --- | --- | --- | --- | --- | --- | --- | --- | --- | --- | --- | --- |
| 1 | ID01_P1_Dead | 20.07.2021 | 1 | 09.12.2021 | 142 | 8228 | 0,96 | 2,4 | 18 | 0,5 | 73,7 | 29,4 | 14,6 | 5,0 | 2,5 |
| 2 | ID02_P2_Alive | 20.07.2021 | 0 | 31.01.2022 | 195 | 5531 | 2,55 | 4,1 | 57 | 1,1 | 97,1 | 37,5 | 13,7 | 7,1 | 2,6 |
| 4 | ID04_P3_Dead | 22.07.2021 | 1 | 21.01.2022 | 183 | 26269 | 2,25 | 4,5 | 17 | 1,1 | 37,1 | 27,1 | 12,1 | 3,1 | 1,4 |
| 5 | ID05_P4_Alive | 22.07.2021 | 0 | 31.01.2022 | 193 | 1512 | 1,48 | 4,8 | 18 | 0,6 | 112,4 | 16,9 | 12,3 | 9,1 | 6,6 |
| 6 | ID06_P5_Alive | 22.07.2021 | 0 | 31.01.2022 | 193 | 5446 | 1,41 | 2,8 | 0,6 | 0,9 | 66,5 | 41,9 | 11,1 | 6,0 | 1,6 |
| 7 | ID07_P6_Alive | 23.07.2021 | 0 | 31.01.2022 | 192 | 2581 | 0,92 | 2,5 | 1,2 | 1,4 | 75,8 | 28,3 | 12,6 | 6,0 | 2,7 |
| 8 | ID08_P7_Alive | 23.07.2021 | 0 | 31.01.2022 | 192 |  | 3,24 | 5,4 | 5,6 | 1,3 | 109,1 | 33,5 | 12,0 | 9,1 | 3,3 |
| 9 | ID09_P8_Alive | 23.07.2021 | 0 | 31.01.2022 | 192 | 1365 |  |  | 201 | 1,8 | 115,5 | 36,8 | 13,6 | 8,5 | 3,1 |
| 10 | ID10_P9_Alive | 23.07.2021 | 0 | 31.01.2022 | 192 | 6587 | 2,29 | 4 | 8 | 1,3 | 83,8 | 29,7 | 12,8 | 6,6 | 2,8 |
| 11 | ID11_P10_Alive | 24.07.2021 | 0 | 31.01.2022 | 191 | 8784 |  |  | 1,9 | 0,9 | 110,4 | 32,5 | 13,9 | 7,9 | 3,4 |
| 12 | ID12_P11_Alive | 27.07.2021 | 0 | 31.01.2022 | 188 | 14088 |  |  | 6,2 | 0,3 | 94,4 | 32,1 | 9,4 | 10,0 | 2,9 |
| 13 | ID13_P12_Alive | 27.07.2021 | 0 | 31.01.2022 | 188 | 22595 | 3 | 4,9 | 16 | 0,6 | 115,2 | 41,7 | 14,4 | 8,0 | 2,8 |
| 14 | ID14_P13_Alive | 27.07.2021 | 0 | 31.01.2022 | 188 | 3785 | 2,09 | 3,3 | 9,3 | 0,9 | 113,9 | 23,6 | 11,9 | 9,6 | 4,8 |
| 15 | ID15_P14_Alive | 27.07.2021 | 0 | 31.01.2022 | 188 | 7985 | 5,2 | 7,7 | 6,8 | 0,5 | 66,5 | 21,8 | 13,9 | 4,8 | 3,0 |
| 16 | ID16_P15_Alive | 27.07.2021 | 0 | 31.01.2022 | 188 | 2958 | 3,53 | 5,2 | 7 | 0,5 | 108,9 | 17,9 | 11,6 | 9,4 | 6,1 |
| 17 | ID17_P16_Dead | 27.07.2021 | 1 | 28.07.2021 | 1 | 1736 | 4,85 | 5,8 | 24 | 1,3 | 65,0 | 24,3 | 13,6 | 4,8 | 2,7 |
| 18 | ID18_P17_Alive | 28.07.2021 | 0 | 31.01.2022 | 187 | 19027 | 2,4 | 4,2 | 31 | 0,2 | 119,7 | 34,2 | 9,8 | 12,2 | 3,5 |
| 19 | ID19_P18_Alive | 30.07.2021 | 0 | 31.01.2022 | 185 | 2738 |  |  | 12 | 1 | 99,4 | 24,5 | 12,2 | 8,1 | 4,1 |
| 20 | ID20_P19_Dead | 02.08.2021 | 1 | 05.01.2022 | 156 | 8558 |  |  | 11 | 0,6 | 60,8 | 22,8 | 10,8 | 5,6 | 2,7 |
| 21 | ID21_P20_Dead | 02.08.2021 | 1 | 20.09.2021 | 49 | 25246 | 1,29 | 2,3 | 31 | 0,5 | 46,9 | 25,3 | 11,1 | 4,2 | 1,9 |
| 22 | ID22_P21_Dead | 02.08.2021 | 1 | 04.08.2021 | 2 | 35000 | 1,46 | 2,9 | 151 | 0,3 | 72,7 | 19,6 | 10,3 | 7,0 | 3,7 |
| 23 | ID23_P22_Alive | 02.08.2021 | 0 | 31.01.2022 | 182 | 4497 | 3,5 | 1,9 | 5,2 | 0,9 | 137,4 | 27,2 | 11,2 | 12,3 | 5,0 |
| 24 | ID24_P23_Alive | 05.08.2021 | 0 | 31.01.2022 | 179 | 3405 | 3,83 | 5,9 | 11 | 1,1 | 78,5 | 28,1 | 10,5 | 7,5 | 2,8 |
| 25 | ID25_P24_Alive | 05.08.2021 | 0 | 31.01.2022 | 179 |  | 3,27 | 5,7 | 2 | 1,2 | 84,9 | 36,1 | 12,4 | 6,9 | 2,4 |
| 26 | ID26_P25_Alive | 05.08.2021 | 0 | 31.01.2022 | 179 | 9674 |  |  | 2,4 | 1,3 | 96,9 | 29,7 | 8,3 | 11,7 | 3,3 |
| 27 | ID27_P26_Dead | 18.08.2021 | 1 | 13.12.2021 | 117 | 35000 | 2,56 | 3,6 | 47 | 0,4 | 97,5 | 31,0 | 11,1 | 8,8 | 3,1 |
| 28 | ID28_P27_Alive | 18.08.2021 | 0 | 31.01.2022 | 166 | 2403 | 1,58 | 3,1 | 6,8 | 1,4 | 105,9 | 27,6 | 9,3 | 11,4 | 3,8 |
| 29 | ID29_P28_Alive | 18.08.2021 | 0 | 31.01.2022 | 166 | 7493 | 1,78 | 3,2 | 6 | 0,9 | 75,8 | 8,4 | 11,2 | 6,8 | 9,1 |
| 30 | ID30_P29_Alive | 19.08.2021 | 0 | 31.01.2022 | 165 | 3677 | 1,47 | 3,5 | 1,1 | 0,9 | 86,4 | 19,1 | 8,6 | 10,1 | 4,5 |
| 32 | ID32_P30_Alive | 23.08.2021 | 0 | 31.01.2022 | 161 | 3191 |  |  | 0,5 | 1,2 | 65,4 | 25,7 | 9,0 | 7,3 | 2,5 |
| 33 | ID33_P31_Alive | 23.08.2021 | 0 | 31.01.2022 | 161 | 2195 |  |  | 0,6 | 0,4 | 65,3 | 32,1 | 11,6 | 5,6 | 2,0 |
| 35 | ID35_P32_Alive | 24.08.2021 | 0 | 31.01.2022 | 160 | 10941 | 2,5 | 4,6 | 16 | 0,9 | 94,4 | 31,8 | 11,4 | 8,3 | 3,0 |
| 36 | ID36_P33_Dead | 27.08.2021 | 1 | 16.09.2021 | 20 | 7185 | 2,89 | 4 | 70 | 0,4 | 39,3 | 33,4 | 8,7 | 4,5 | 1,2 |
| 37 | ID37_P34_Alive | 27.08.2021 | 0 | 31.01.2022 | 157 | 3498 |  |  | 16 | 1 | 139,9 | 24,9 | 10,1 | 13,9 | 5,6 |
| 38 | ID38_P35_Dead | 27.08.2021 | 1 | 02.12.2021 | 97 | 11873 | 1,21 | 3,1 | 6,2 | 0,4 | 77,8 | 28,4 | 8,2 | 9,5 | 2,7 |
| 39 | ID39_P36_Alive | 31.08.2021 | 0 | 31.01.2022 | 153 | 2113 | 3,42 | 4,9 | 61 | 1 | 129,3 | 28,9 | 9,7 | 13,3 | 4,5 |
| 40 | ID40_P37_Dead | 31.08.2021 | 1 | 02.12.2021 | 93 | 35000 |  |  | 18 | 0,1 | 107,3 | 26,7 | 8,6 | 12,4 | 4,0 |
| 41 | ID41_P38_Dead | 31.08.2021 | 1 | 08.09.2021 | 8 | 3008 | 1,93 | 2,9 | 31 | 0,5 | 128,6 | 20,6 | 11,5 | 11,2 | 6,2 |
| 42 | ID42_P39_Alive | 31.08.2021 | 0 | 31.01.2022 | 153 | 5197 |  |  | 158 | 1,5 | 67,3 | 43,9 | 10,0 | 6,8 | 1,5 |
| 43 | ID43_P40_Alive | 02.09.2021 | 0 | 31.01.2022 | 151 | 1020 | 2,81 | 4,1 | 2,3 | 1,4 | 64,7 | 24,0 | 8,9 | 7,2 | 2,7 |
| 46 | ID46_P41_Alive | 02.09.2021 | 0 | 31.01.2022 | 151 | 11591 | 1,91 | 3 | 0,5 | 1,5 | 60,0 | 24,8 | 8,0 | 7,5 | 2,4 |
| 47 | ID47_P42_Alive | 02.09.2021 | 0 | 31.01.2022 | 151 | 8634 | 1,6 | 2,4 | 13 | 1,1 | 109,1 | 22,0 | 10,1 | 10,8 | 5,0 |
| 48 | ID48_P43_Alive | 03.09.2021 | 0 | 31.01.2022 | 150 | 2203 | 1,74 | 3,6 | 1,7 | 1,4 | 80,0 | 21,3 | 9,0 | 8,9 | 3,8 |
| 49 | ID49_P44_Alive | 03.09.2021 | 0 | 31.01.2022 | 150 | 1437 | 3 | 4,2 | 8 | 1,6 | 80,6 | 18,4 | 9,9 | 8,2 | 4,4 |
| 52 | ID52_P45_Dead | 07.09.2021 | 1 | 03.12.2021 | 87 | 1852 | 1,27 | 3,1 | 0,5 | 1,2 | 81,1 | 23,4 | 8,3 | 9,8 | 3,5 |
| 53 | ID53_P46_Alive | 07.09.2021 | 0 | 31.01.2022 | 146 | 2513 | 2,44 | 5,4 | 0,5 | 0,7 | 130,4 | 23,6 | 7,5 | 17,4 | 5,5 |
| 54 | ID54_P47_Alive | 07.09.2021 | 0 | 31.01.2022 | 146 | 12970 | 2,63 | 3,5 | 4,9 | 1,2 | 95,3 | 31,8 | 7,2 | 13,2 | 3,0 |
| 55 | ID55_P48_Dead | 07.09.2021 | 1 | 30.12.2021 | 114 | 11263 |  |  | 57 | 0,6 | 108,8 | 26,5 | 7,4 | 14,7 | 4,1 |
| 57 | ID57_P49_Alive | 07.09.2021 | 0 | 31.01.2022 | 146 | 2014 | 1,85 | 3,9 | 13 | 0,6 | 96,4 | 31,3 | 9,2 | 10,5 | 3,1 |
| 58 | ID58_P50_Alive | 07.09.2021 | 0 | 31.01.2022 | 146 | 8448 | 1,63 | 3,6 | 1,1 | 0,5 | 131,0 | 24,5 | 6,5 | 20,0 | 5,4 |

**Table S2-S9**

Separate Supplementary files in Excel format.

**Table S10.** Results from Cox regression analysis

| Table Analyzed | Data |  |  |  |
| --- | --- | --- | --- | --- |
| Time variable | Days of survival |  |  |  |
| Censor/Event variable | Death during follow-up period (1-yes, 0-no) | | |  |
| Regression type | Cox regression |  |  |  |
| Estimation method | Exact |  |  |  |
|  |  |  |  |  |
| Model |  |  |  |  |
| Parameter estimates | Variable | Estimate | Standard error | 95% CI (profile likelihood) |
| β1 | ln(NT-proBNP (ng/l)) | -0.2755 | 0.4297 | -1.153 to 0.5637 |
| β2 | ln(CRP (mg/l)) | 0.4478 | 0.2420 | -0.009244 to 0.9558 |
| β3 | ln(eGFR (ml/s/1.73m2)) | -0.9425 | 0.5098 | -1.980 to 0.05910 |
| β4 | ln(PC 42:10) | -2.850 | 0.9592 | -4.834 to -1.001 |
|  |  |  |  |  |
| Hazard ratios | Variable | Estimate | 95% CI (profile likelihood) |  |
| exp(β1) | ln(NT-proBNP (ng/l)) | 0.7592 | 0.3156 to 1.757 |  |
| exp(β2) | ln(CRP (mg/l)) | 1.565 | 0.9908 to 2.601 |  |
| exp(β3) | ln(eGFR (ml/s/1.73m2)) | 0.3896 | 0.1381 to 1.061 |  |
| exp(β4) | ln(PC 42:10) | 0.05783 | 0.007953 to 0.3674 |  |
|  |  |  |  |  |
| Sig. diff. than zero? | Variable | \|Z\| | P value | P value summary |
| β1 | ln(NT-proBNP (ng/l)) | 0.6412 | 0.5214 | ns |
| β2 | ln(CRP (mg/l)) | 1.850 | 0.0643 | ns |
| β3 | ln(eGFR (ml/s/1.73m2)) | 1.849 | 0.0645 | ns |
| β4 | ln(PC 42:10) | 2.971 | 0.0030 | ** |
